# Necessity of fusion following decompression surgery in patients with single-level lumbar stenosis: study protocol for an open-label multicentre non-inferiority randomized controlled clinical trial

**DOI:** 10.1186/s13063-023-07486-8

**Published:** 2023-07-10

**Authors:** Andrey Grin, Ivan Lvov, Aleksandr Talypov, Vladimir Smirnov, Anton Kordonskiy, Valeriy Lebedev, Andrey Zuev, Ilya Senko, Iren Pogonchenkova, Vladimir Krylov

**Affiliations:** 1 Sklifosovsky Research Institute for Emergency Medicine, Moscow, Russia; 2 Pirogov National Medical and Surgical Center, Moscow, Russia; 3 Federal State Budgetary Institution “Federal Center for Brain and Neurotechnologies” FMBA, Moscow, Russia; 4 Moscow Scientific and Practical Center for Medical Rehabilitation, Restorative and Sports Medicine, Moscow, Russia

**Keywords:** Randomized controlled trial, Lumbar spinal stenosis, Laminotomy, Spinal Fusion, Decompression

## Abstract

**Background:**

The necessity of spinal segment fusion after decompression is one of the most controversial and unresolved issues in single-level lumbar spinal stenosis surgery. To date, only one trial carried out 15 years ago focused on this problem. The key purpose of the current trial is to compare the long-term clinical results of the two surgical methods (decompression vs. decompression and fusion) in patients with single-level lumbar stenosis.

**Methods:**

This study is focused on the non-inferior clinical results of decompression compared with the standard fusion procedure. In the decompression group, the spinous process, the interspinous and supraspinous ligaments, part of the facet joints, and corresponding parts of the vertebral arch are to be preserved intact. In the fusion group, decompression is to be supplemented with transforaminal interbody fusion. Participants meeting the inclusion criteria will be randomly divided into two equal groups (1:1), depending on the surgical method. The final analysis will include 86 patients (43 per group). The primary endpoint is Oswestry Disability Index dynamics at the end of the 24-month follow-up compared to the baseline level. Secondary outcomes included those estimated using the SF-36 scale, EQ-5D-5L, and psychological scales. Additional parameters will include sagittal balance of the spine, fusion results, total cost of surgery, and hospital stay followed by two-year treatment. Follow-up examinations will be performed at 3, 6, 12, and 24 months

**Discussion:**

Authors suggest that this study will improve the evidence for application of various surgical techniques for lumbar spine stenosis surgery and verify the existing protocol for surgical management.

**Trial registration:**

ClinicalTrials.gov NCT05273879. Registered on March 10, 2022.

**Supplementary Information:**

The online version contains supplementary material available at 10.1186/s13063-023-07486-8.

## Administrative information

Note: the numbers in curly brackets in this protocol refer to SPIRIT checklist item numbers. The order of the items has been modified to group similar items (see http://www.equator-network.org/reporting-guidelines/spirit-2013-statement-defining-standard-protocol-items-for-clinical-trials/).

**Table:** 

Title {1}	Necessity of fusion following decompression surgery in patients with single-level lumbar stenosis: study protocol for an open-label multicenter non-inferiority randomized controlled clinical trial
Trial registration {2a and 2b}.	ClinicalTrials.gov NCT05273879 https://clinicaltrials.gov/ct2/show/NCT05273879
Protocol version {3}	Version 2 08.09.2022.
Funding {4}	Provided study will be funded from the local budgets of all participating hospitals.
Author details {5a}	1) Andrey Grin. Prof., MD, PhD. Sklifosovsky Research Institute for Emergency Medicine, Moscow, Russia 2) Ivan Lvov. MD, PhD. Sklifosovsky Research Institute for Emergency Medicine, Moscow, Russia 3) Aleksandr Talypov. Prof., MD, PhD Sklifosovsky Research Institute for Emergency Medicine, Moscow, Russia 4) Vladimir Smirnov. MD, PhD. Sklifosovsky Research Institute for Emergency Medicine, Moscow, Russia 5) Anton Kordonskiy. MD, PhD. Sklifosovsky Research Institute for Emergency Medicine, Moscow, Russia 6) Valeriy Lebedev. MD, PhD. Pirogov National Medical and Surgical Center, Moscow, Russia 7) Andrey Zuev. MD, PhD. Pirogov National Medical and Surgical Center, Moscow, Russia 8) Ilya Senko. MD, PhD. Federal State Budgetary Institution "Federal Center for Brain and Neurotechnologies" FMBA, Moscow, Russia 9) Iren Pogonchenkova. MD, PhD. Moscow Scientific and Practical Center for Medical Rehabilitation, Restorative and Sports Medicine, Moscow, Russia 10) Vladimir Krylov. Prof., MD, PhD Sklifosovsky Research Institute for Emergency Medicine, Moscow, Russia.
Name and contact information for the trial sponsor {5b}	No sponsor
Role of sponsor {5c}	No sponsor

## Introduction

### Background and rationale {6a}

Degenerative lumbar disease is one of the most common chronic diseases worldwide. The general incidence of lumbar stenosis accompanied by a significant deterioration in the quality of life reaches 5% among patients aged < 50 years and approximately 10–15% among elderly patients (50–70 years old). Moreover, lumbar stenosis appears to be one of the most common causes of decompression and fusion interventions in the lumbar spine in 50+ years old patients [1]. Applicable surgery includes the removal of bone and ligamentous structures compressing the cauda equina roots inside the spinal canal. The necessity for significant facet joint resection results in instability of the corresponding spinal segment and/or kyphotic deformation, leading to an obligatory subsequent interbody fusion.

One of the most controversial and unresolved problems of current degenerative spine management is the necessity of spinal segment fusion in patients with grade C and D lumbar stenosis according to the Schizas classification [2] without any radiological signs of instability. The decompression alone method is associated with significantly lower 2-year payments when treating stenosis [3]. Furthermore, the meta-analysis conducted by Ma et al. [4] reported a lower number of complications in the decompression-only patients’ group, while the fusion method demonstrated greater clinical improvement. According to the meta-analysis conducted by Shen et al. [5], the decompression method was associated with a shorter hospital stay, but a greater shift in the mean difference value for clinical improvement was observed in favour of the fusion group. It is important to note that both of these meta-analyses had limitations due to the inclusion of patients with lumbar spondylolisthesis, which could have a significant impact on the clinical outcomes.

To date, we have found only one randomized controlled trial [6] that specifically focuses on stenosis without spondylolisthesis and utilizes modern methods of minimally invasive surgery. The authors have not found superiority of fusion surgery for patients with lumbar spine stenosis. This study was initiated 15 years ago and had several limitations, including a high level of group heterogeneity (for example, in lumbar stenosis severity) and a wide range of surgical techniques. Moreover, the authors did not address the impact of postoperative rehabilitation treatment on long-term clinical outcomes. In addition, the authors did not consider sagittal imbalance of the spine, which could have a significant impact on treatment outcomes.

Thus, considering the lower number of complications, reduced hospital stay, and economic efficiency of decompression without fusion, while demonstrating non-inferior clinical efficacy compared to fusion and non-fusion techniques, this trial could offer surgeons a rationale for choosing the less invasive treatment option.

### Objectives {7}

The objective is to compare the long-term clinical outcomes of two surgery methods (decompression versus decompression and fusion) in patients with single-level lumbar stenosis.

### Trial design {8}

This study is an open-label, non-inferior, multicentre, randomized controlled trial. All enrolled subjects will be divided into two concurrent groups based on the surgical technique applied (decompression or decompression with fusion).

#### Patient and public involvement

Patients were not involved in the design of this research.

## Methods: participants, interventions, and outcomes

### Study setting {9}

This study involves five surgeons from three independent centres performing surgery on patients with degenerative spines. These included Sklifosovsky Research Institute for Emergency Medicine and Federal Center for Brain and Neurotechnologies as major centres and Pirogov’s National Medical and Surgical Center as a level 3 trauma centre. All participating spine surgeons are highly experienced (more than 12 years) in surgery for degenerative spine diseases and perform at least 180 operations annually. Both surgical techniques (decompression and decompression with fusion) have been used in all three clinical centres for more than 10 years. Fusion is routinely used in cases of instability that are confirmed radiologically or intraoperatively. The decompression technique was selected based on the surgeon’s personal opinion. However, all surgeons are highly experienced in both types of surgery.

In all cases, rehabilitation treatment is to be carried out by Branch No. 3 of the Moscow Scientific and Practical Center for Medical Rehabilitation, Restorative and Sports Medicine. Three experienced rehabilitation specialists will participate in the study. To avoid postoperative treatment heterogeneity, they will use a unified protocol for the postoperative treatment of all patients in the study

### Eligibility criteria {10}

Primary patient selection will be performed before admission. The study will include participants aged 45–75 years with symptomatic single-level stenosis at the level of L2-L3, L3-L4, L4-L5, or L5-S1 vertebrae. The inclusion and exclusion criteria are presented in Table 1. Patients who meet the inclusion criteria but refuse to participate in the study will be marked with an appropriate explanation.

**Table 1:** Inclusion and exclusion criteria

Inclusion criteria:
- Age ranging from 45 to 75 years
- C or D grade lumbar stenosis at the level of L2-L3, L3-L4, L4-L5, or L5-S1 vertebrae (classification by Shizas et al.) according to MRI evaluation
- A clinical manifestation of lumbar stenosis (neurogenic claudication syndrome and/or radiculopathy)
- Insufficient conservative therapy within 3 months prior to surgery
- Informed consent was obtained from all included subjects
Exclusion criteria:
- Spondylolisthesis exceeding 3 mm
- Spinal instability according to functional radiography
- Sagittal imbalance (type 4 according to C. Barrey)
- Bone density of the vertebrae at the surgery level below 100 HU
- Clinically significant multi-level spinal stenosis (two or more segments)
- Previously performed surgeries at the lumbar level
- Severe sacroiliac or lumbar facet joint pain
- Risk of anaesthesia exceeding four or five grades according to ASA
- Inability to participate in control examinations within 2 years, postoperatively
- Participation in other clinical trials related to surgical or conservative treatment of spine diseases

**Fig. 1:**
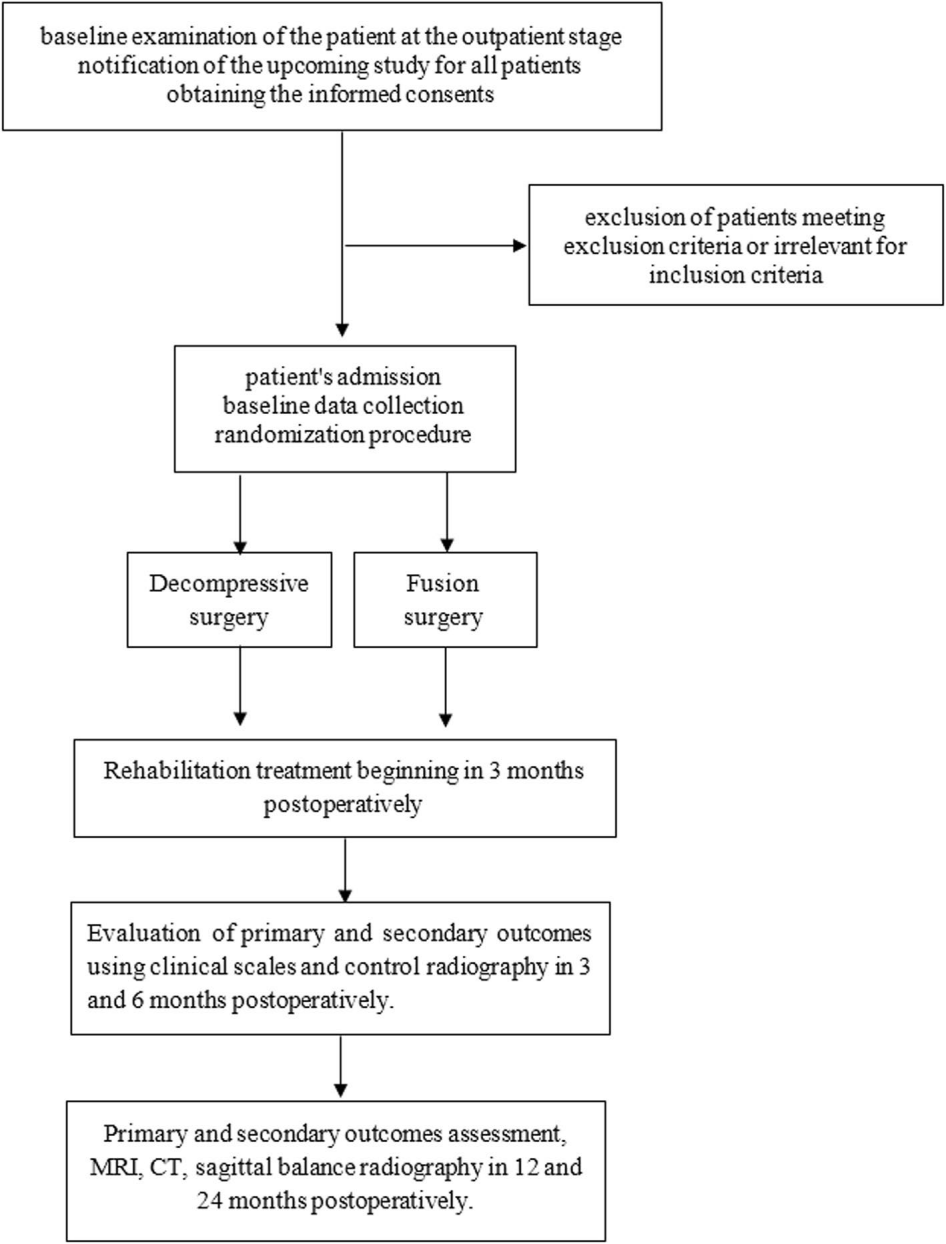
Flow chart

A flow chart of the trial is provided in Fig. 1.

### Who will take informed consent? {26a}

The consultant neurosurgeon of outpatient clinic will take informed consent during consultation meeting with the patient that is eligible for inclusion.

### Additional consent provisions for collection and use of participant data and biological specimens {26b}

All patients will be informed about the upcoming study. Written and oral informed consent is required prior to the randomization procedure. Study information is given by the consultant neurosurgeon. No biological specimens will be collected in this trial.

### Interventions

#### Explanation for the choice of comparators {6b}

Combination of decompression and fusion or decompression alone are the most widely used surgical *options* for Schizas grade C or D stenosis. Investigators suggest that efficiency of decompression alone technique is comparable to the one of lumbar fusion in patients with lumbar stenosis

#### Intervention description {11a}

All surgical procedures are to be performed under general endotracheal anaesthesia.

In the decompression group, laminotomy of the corresponding adjacent vertebrae, partial flavectomy, and medial facetectomy are planned to be performed unilaterally. Depending on the surgeon’s personal preferences, the following two options are available: (1) equivalent decompression procedure contralaterally and (2) crossover contralateral decompression. Irrespective of the selected option, the spinous process, the interspinous and supraspinous ligaments, part of the facet joints, and the corresponding part of the vertebral arch must be preserved intact in all participants.

In the fusion group, the surgical course will consist of two stages: (a) decompression performed using one of the above-mentioned methods followed by (b) transforaminal interbody fusion using cage (TLIF) and fixation using pedicle screws.

#### Criteria for discontinuing or modifying allocated interventions {11b}

Patients are allowed to terminate their participation in the trial at any moment. The principal investigator may withdraw a patient’s participation for any reason in case of conflict of patient’s interest.

#### Strategies to improve adherence to interventions {11c}

In each case, MRI images and examination data are reviewed and discussed by the study group. If necessary, an additional discussion is carried out comprising the patient and all operating surgeons.

#### Relevant concomitant care permitted or prohibited during the trial {11d}

Within 3 months postoperatively, all patients will undergo a course of rehabilitation treatment. This treatment will be conducted by the same specialists and according to unified programs. Patients are prevented from participating in other randomized clinical trials.

#### Provisions for post-trial care {30}

All enrolled subjects have standard state insurance for non-negligent harm. Additional health care, compensation, or damages will be provided by participating clinical centres.

## Outcomes {12}

### Primary endpoint

The primary endpoint includes the dynamics of ODI parameters at the end of the 24-month follow-up compared to the baseline level [7]. This patient-reported measurement precisely estimates the impairment of a participant’s quality of life resulting from low back pain and clearly reflects the clinical outcome of the treatment. Parameter assessment is to be considered at all stages of the study beginning from baseline examination, followed by a follow-up examination 2 years postoperatively. The questionnaire consists of 10 sections, comprising six statements per section. Each answer is assessed using six grades (ranging from 0 to 5) with a maximum score of 50 points per participant. Subsequently, the total score is converted into a percentage ratio (from 0 to 100%). If any of the sections are not applicable or have been withdrawn for ethical reasons, the total score is calculated within nine sections and is divided by 45.

### Secondary endpoints

The SF-36 v.1 scale (standard form) provides a comprehensive evaluation of the quality of life both pre- and postoperatively [8]. The SF-36 questionnaire consists of 36 questions grouped into eight sections assessing physical functioning, role functioning, bodily pain, general health condition, vitality, social functioning, emotional state, and mental health. The maximum score of each section is limited to 100 points. These eight scales will be aggregated into two summary measures: physical (PH) and mental (MH) health summary scores.

Another scale assessing participants’ quality of life is the EQ-5D-5L [9]. This questionnaire is widely used in multiple prospective European studies focusing on lumbar stenosis treatment. Currently, we believe this scale is applicable for comparing our results with literature data. The EQ-5D-5L questionnaire consists of five sections (mobility, self-care, usual activities, pain, and anxiety) comprising five levels per section, supplemented with the EQ-VAS visual analogue scale. The obtained results can be easily converted into a single numerical value (index) adjusted for the patient’s region of residence. Currently, there are no approved values for calculating this index in the Russian Federation (https://euroqol.org). Therefore, at baseline, a simple comparison of the specific numerical values for each parameter will be applied. If feasible, we will calculate this index at the end of the study.

The Won-Korff Chronic Pain Syndrome Assessment Scale (Chronic Pain Grade Questionnaire, CPGQ) [10] is focused on the assessment of pain severity and its impact on participants’ quality of life. Grade 0 corresponds to the absence of pain, while grade IV corresponds to a severe decrease in the quality of life due to severe pain.

The Pain Catastrophizing Scale (PCS) [11] was created to estimate the psychological aspects of pain perception, attenuating the exaggerated negative perception of painful stimuli. The questionnaire contains 13 questions assessed in points (ranging from 0 points (no symptoms) to 4 points (negative perception is experienced constantly).

The Chronic Pain Coping Inventory (CPCI) [12] includes 64 questions, allowing one of eight alternative scales to differentiate between participants’ preferred strategies for managing chronic pain.

Preoperatively, the physical status of all included participants will be assessed using the American Society of Anesthesiologists (ASA) scale. This classification identifies five classes of physical status depending on the presence of concomitant diseases (ranging from class I (healthy patient) to class V (dying).

In addition, the total cost of surgery and hospital stay will be calculated based on the cost of surgical intervention and inpatient treatment and the indirect cost of patients’ occupational disability. Two years postoperatively, the cost of subsequent treatment in the rehabilitation centre, additional treatment of possible postoperative complications, and/or treatment of other manifestations of degenerative spine disease as well as period of incapacity for work are to be considered.

All participants who have undergone spine instrumentation will be examined for intervertebral fusion parameters using the criteria of Tan et al. [13]; grade 1 corresponds to complete bone union, whereas grade 4 corresponds to pseudarthrosis. Moreover, the time of fusion development will be estimated.

Finally, sagittal balance parameters will be analysed in all patients, including pelvic tilt (PI), pelvic deviation from the vertical (PT), S1 vertebral tilt (SS), and vertical axis displacement (SVA).

#### Participant timeline {13}

The study coordinators in all involved clinical centres are responsible for data collection and administration at baseline and during follow-up. All examinations (questionnaires, scales, radiography, and CT) will be performed during personal follow-up visits at the original centre of surgery performance. All the obtained data will be stored in the data centre of the Sklifosovsky Research Institute for Emergency Medicine. These data will be unavailable to all parties until the final analysis. The timetable for data collection is presented in Table 2.

**Table 2:** Timetable for data collection

	Preoperatively	Inpatient stay	3 mo p/o	6 mo p/o	12 mo p/o	24 mo p/o
Demographics, lifestyle, clinical data	+					
Informed consent	+					
Clinical measures						
ODI	+		+	+	+	+
SF-36	+		+	+	+	+
EQ-5D-5L	+		+	+	+	+
ASA	+					
Psychological measures						
CPGQ	+		+	+	+	+
PCS	+		+	+	+	+
CPCI	+		+	+	+	+
Radiography of the lumbar spine		+	+	+		
Radiograph sagittal balance assessment	+				+	+
CT	+				+	+
MRI	+				+	+
Randomization		+				
Admittance and surgery data		+				
Cost of surgical treatment and hospital stay		+				
Cost of rehabilitation treatment			+			
Cost of treatment in other clinical facilities			+	+	+	+
Period of incapacity for work	+		+	+	+	+
Complications, repeated surgeries		+	+	+	+	+
Follow-up visits and other healthcare facilities			+	+	+	+

*ASA* American Society of Anesthesiologists Scale, *CPGQ* Chronic Pain Grade Questionnaire, *CPCI* Chronic Pain Coping Inventory; *CT*, computed tomography, *EQ-5D-5L* EuroQol five-dimensional five-level descriptive system questionnaire, *MRI* Magnetic resonance imaging, *ODI* Oswestry Disability Index, *PCS* Pain Catastrophizing Scale, *p/o* Postoperatively, *SF-36* Short Form-36 questionnaire, *mo* month

#### Sample size {14}

Following a non-inferiority study design, we calculated the sample size based on the primary outcome. According to the most recent randomized trial [6], the standard deviation for the ODI in the fusion group is 20 points. With a non-inferiority margin of δ = 12, two-sided α-level of 0.05, power of 80%, and 1:1 allocation ratio, the required sample size is 70 patients. Considering the potential of 20% of patients missing the obligatory follow-up procedures, we plan to include at least 86 patients (43 patients per group).

#### Recruitment {15}

Both participating clinical centres perform more than 1 000 spinal surgeries per year. Among these, more than 80 patients with single-level lumbar stenosis met the inclusion criteria for the study. We expect that up to 40% of patients provide their informed consent to participate in randomization. Consequently, we suggest to reach the required sample size within 2 years.

## Assignment of interventions: allocation

### Sequence generation {16a}

All participants who meet the inclusion criteria and provide informed consent will be randomly divided into two equal groups (1: 1) according to the applied surgical technique. For the block randomization procedure, stratification based on the severity of lumbar stenosis (Schizas C and D) will be performed. Randomization will be by random permuted blocks of 4 and 6.

### Concealment mechanism {16b}

Participant allocation will be conducted using sequentially numbered, opaque and sealed envelopes, opened only at the time of participant’s inclusion in the study.

### Implementation {16c}

Randomization will be provided one day preoperatively by an independent remote study team member excluded from any other study roles. Randomization parameters will be documented in the participants’ records.

## Assignment of interventions: blinding

### Who will be blinded {17a}

Not applicable, no blinding.

### Procedure for unblinding if needed {17b}

The design of the study is open label with only outcome assessors being blinded, so unblinding will not occur.

## Data collection and management

### Plans for assessment and collection of outcomes {18a}

The study coordinators in all involved clinical centres are responsible for data collection and administration at baseline and during follow-up. All examinations (questionnaires, scales, radiography, and CT) will be performed during personal follow-up visits at the original centre of surgery performance.

### Plans to promote participant retention and complete follow-up {18b}

Communication with patients will be carried out via e-mail in accordance with the approved schedule (Table 2). If after 3 letters the patient does not get in touch, a phone call will be made. If the patient is not found within 2 weeks after the date of the follow-up visit, he will be excluded from the study.

### Data management {19}

All the obtained data will be stored in the data centre of the Sklifosovsky Research Institute for Emergency Medicine. These data will be unavailable to all parties until the final analysis.

### Confidentiality {27}

Each patient will be assigned a unique number; only the investigator team has access to the ID of the recruited participants.

### Plans for collection, laboratory evaluation, and storage of biological specimens for genetic or molecular analysis in this trial/future use {33}

Not applicable; no biological sampling nor laboratory investigation is planned for this study.

## Statistical methods

### Statistical methods for primary and secondary outcomes {20a}

The results will be analysed using the intention-to-treat principle. Statistical significance will be defined as *p* < 0.05 based on a two-sided test. No adjustments for multiple tests are planned. The normality of variables will be evaluated using the Shapiro-Wilk statistic. Student’s *t*-test (for normally distributed data) and Pearson’s chi-square test (for categorical data) will be used to reveal differences in symptom rates among the groups. Multiple linear regression will be used to measure point estimates and confidence intervals for group differences in the ODI and clinical scale scores from baseline to the 2-year follow-up.

The non-parametric Mann-Whitney *U* test will be used to reveal differences in continuous variables between groups both pre- and postoperatively. Continuous data within the groups will be compared using the Wilcoxon test. Categorical parameters among the groups will be compared using Fisher’s exact two-tailed test.

Complication incidence during the 2-year follow-up will be compared between groups using Kaplan–Meier survival curves and the log-rank test. The significance of the risk factors (predictors) for all registered complications will be evaluated using univariate and multivariate logistic regression models.

### Interim analyses {21b}

Interim analysis of the obtained follow-up data will be available by 12 months postoperatively for at least 20 patients in each cohort. The criteria for study termination include the following: (1) the number of repeated surgeries in any group exceeds 50%, (2) any group demonstrates a higher frequency of negative neurological postoperative outcomes and/or significant decline of ODI level compared to baseline by at least 10 points, and (3) the number of postoperative complications exceeding 50% in any group.

An interim analysis will be performed by an investigator not involved in patient treatment (G. Kireeva, MD, PhD).

### Methods for additional analyses (e.g., subgroup analyses) {20b}

No additional analyses are planned.

### Methods in analysis to handle protocol non-adherence and any statistical methods to handle missing data {20c}

For missing outcome data, multiple imputations will be used.

### Plans to give access to the full protocol, participant level-data and statistical code {31c}

The datasets analysed during the current study and statistical code are available from the corresponding author on reasonable request, as is the full protocol.

## Oversight and monitoring

### Composition of the coordinating centre and trial steering committee {5d}

The coordinating centre is Sklifosovsky Research Institute for Emergency Medicine. The steering committee include:

- Chief investigator—prof. A. Grin, MD, PhD has overall supervision of study;
- Researchers—V. Smirnov, MD, PhD, A. Kordonskiy, MD, PhD Responsible for recruitment, data collection, adherence to study protocol;
- Critical reviewer—prof. A. Konovalov, MD, PhD (Burdenko Neurosurgical Center, Moscow, Russia);
- Independent observer—G. Kireeva, MD, PhD (Pirogov National Medical and Surgical Center, Moscow, Russia). Responsible for interim analysis and auditing trial conduct.

Each clinical centre provide a local coordinator staying in direct contact with the chief investigator and the research group.

### Composition of the data monitoring committee, its role and reporting structure {21a}

Data monitoring is performed to ensure that all events (i.e. PROMS, radiology) are performed according to the study protocol. The data monitoring committee consists of the chief investigator (A. Grin, Prof., MD, PhD) and researchers (A. Talypov, MD, PhD, and I. Lvov, MD, PhD). Other investigators are responsible for recruitment, data collection, adherence to study protocol: S. Zuev, MD, E. Sosnovskiy, MD, PhD, D. Epifanov, MD, A. Kalandari, MD, PhD)

### Adverse event reporting and harms {22}

Potentially analysed adverse events (AE) include implant failure, infection, neurologic deterioration after surgery, and other implant- or approach-related complications. Other AEs are not considered, including pulmonary embolism due to deep vein thrombosis, sepsis, meningitis, severe heart failure, etc. All AEs are reported directly to the chief investigator.

### Frequency and plans for auditing trial conduct {23}

Study-related monitoring, audits, and inspections of all documents by the trial steering group, the data monitoring committee, and ethics board are planned prior to inclusion of the first patient (baseline), then every 20 inclusions (intermediate) and after the final inclusion (final).

### Plans for communicating important protocol amendments to relevant parties (e.g. trial participants, ethical committees) {25}

All potential changes for the RCT’s protocol will be reported and resubmitted for approval, that is, equal to changes in objectives, endpoints, design, participants population, sample sizes, procedures, or significant administrative aspects.

### Dissemination plans {31a}

Study results will be provided to peer-reviewed neurosurgical and spine surgery journals and presented at international conferences.

## Discussion

Currently, the selection of surgical solutions for lumbar stenosis remains controversial. This study was designed to provide surgeons with high-level evidence by comparing the clinical outcomes of the two most popular treatment methods. The non-inferiority study design [14] was chosen because according to the current evidence [4, 5] and our experience, both methods are non-inferior (comparable) in terms of the primary endpoint, while there may be superiority in certain secondary endpoints, such as cost effectiveness and lower complication rates.

The non-inferior margin was chosen based on the minimum clinically important difference (MCID), which represents the smallest improvement considered worthwhile by a patient. For patients with lumbar spine stenosis, the MCID in ODI score ranges from 12.54 to 12.8 points [15, 16]. Therefore, a non-inferiority margin of *δ* = 12 was selected according to this data.

To develop the structure of this study, we did our best to maximise the homogeneity of the participant groups. For this purpose, we used precise inclusion and exclusion criteria and accurately restricted the range of surgical methods and postoperative rehabilitation treatment. The key role of postoperative rehabilitation in favourable clinical outcomes is widely known. In our study, all included participants are scheduled for rehabilitation treatment at the same rehabilitation centre within 3 months postoperatively. We believe that these conditions maximise the homogeneity of the participant groups and limit possible clinical outcomes by several factors. We suggest that the mentioned structure of the study implicates decompressive intervention as the only factor for favourable clinical outcomes compared to spinal fusion.

In addition to clinical outcomes, we intend to analyse the economic aspects of lumbar stenosis treatment during both in-hospital stay and two postoperative years. Most of the available published studies did not provide any information concerning the cost of treatment and monitoring results. In most developed countries, the cost of treatment may be determined by insurance data and vary significantly. In Russia, all enrolled participants have mandatory medical insurance and implied unified tariffication of all postoperative medical procedures during the 2-year follow-up period. This study might clarify the exact factors, both clinical and financial, to avoid “preventive” fusion in such patients. This can also have a considerable impact on potential outcomes compared with other treatment alternatives

## Trial status

Protocol version: V.2 08.09.2022 Recruiting from April 2022. Approximate date of recruitment completion—December 2024

## Supplementary Information

**Additional file 1.** English and Russian versions of the informed consent.

## Data Availability

All data generated or analysed during the study are included to the published article.

## Abbreviations

ASA: American Society of Anesthesiologists
CPGQ: Chronic Pain Grade Questionnaire
CPCI: Chronic Pain Coping Inventory
CT: Computed tomography
EQ-5D-5L: EuroQol five-dimensional five-level descriptive system questionnaire
Mo: Month
MRI: Magnetic resonance imaging
ODI: Oswestry Disability Index
PCS: Pain Catastrophizing Scale
p/o: Postoperatively
SF-36: Short Form-36 questionnaire
SPIRIT: Standard Protocol Items: Recommendations for Interventional Trials
VAS: Visual analogue scale
