# Supplementary material for: Necessity of fusion following decompression surgery in patients with single-level lumbar stenosis: study protocol for an open-label multicentre non-inferiority randomized controlled clinical trial
Source: Trials. 2023 Jul 10;24:451. doi: 10.1186/s13063-023-07486-8 (PMC10332049; doi:10.1186/s13063-023-07486-8)
Supplement: Supplementary file 1 — Additional file 1. English and Russian versions of the informed consent. [file 13063_2023_7486_MOESM1_ESM.pdf]

НИИ СКОРОЙ ПОМОЩИ им. Н.В. СКЛИФОСОВСКОГО

Необходимость выполнения стабилизации позвоночника после декомпрессии невральных структур у пациентов с одноуровневыми стенозами позвоночного канала на поясничном уровне

Открытое рандомизированное контролируемое исследование, направленное на сравнение отдаленных клинических результатов применения двух методов хирургического лечения (декомпрессия позвоночного канала с или без проведения спондилодеза) у пациентов с одноуровневым стенозом позвоночного канала на поясничном уровне

№ исследования: 1-22/11.01.22

Регистрационный № исследования: NCT05273879

Дата и версия протокола: Версия 2 от 08.09.22

Главный исследователь: проф. А.А. Гринь

Участвующие учреждения:

1) ГБУЗ НИИ СП им. Н.В. Склифосовского ДЗМ;

2) ФГБУ «НМХЦ им. Н.И. Пирогова» Минздрава России;

3) ФБГУ «Федеральный центр мозга и нейротехнологий» ФМБА;

4) ГАУЗ «Московский научно-практический центр медицинской реабилитации, восстановительной и спортивной медицины ДЗМ»

Оглавление

1 Сокращения ... 4
2 Обоснование исследования ... 5
2.1 Вступление ... 5
2.2 Цель исследования ... 7
3 Оценка клинических исходов ... 8
3.1 Оценка первичного клинического исхода ... 8
3.2 Оценка вторичных клинических исходов ... 8
4 участники исследования ... 11
5 Дизайн исследования и его регистрация ... 12
6 Отбор пациентов и описание операций ... 13
6.1 Участники исследования ... 13
6.2 Отбор пациентов ... 14
6.3 Методы хирургического лечения ... 17
6.4 Процедура рандомизации ... 17
6.5 Ослепление ... 18
7 Сбор данных ... 19
8 Статистика ... 21
8.1 Расчет выборки пациентов ... 21
8.2 Статистический анализ ... 21
9 Промежуточный анализ и прекращение исследования ... 23
10 Этические аспекты исследования ... 24
11 Финансирование ... 25
12 Список литературы ... 26
Приложение 1. Информация для пациента и согласие на участие в исследовании ... 28
Часть 1. Информационный блок ... 28
Часть 2. Информированное согласие на участие в исследовании 36
Приложение 2. Описание шкал, применяемых в исследовании ... 37
SF-36 ... 37
Шкала Oswestry ... 40
EQ-5D-5L ... 42
Шкала боли VAS ... 44

Шкала совладания с болью 45
Опросник Вон Корффа..47
Шкала катастрофизации боли 48

1 Сокращения

ASA – Американская Ассоциация Анестезиологов

CONSORT - Consolidated Standards of Reporting Trials, сводные стандарты отчетов в исследованиях

CPGQ - шкала оценки хронического болевого синдрома Вон-Корффа

CPCI - опросник совладания с болью

EQ-5D-5L - EuroQol five-dimensional five-level descriptive system questionnaire

ODI – шкала Освестри

PCS - шкала катастрофизации боли

SF-36 - Short Form-36 questionnaire

SPIRIT - стандартный рекомендательный протокол для интервенционных исследований

VAS – визуально-аналоговая шкала

КТ – компьютерная томография

Мес - месяцы

МРТ – Магнитно-резонансная томография

п/о - послеоперационный

2 Обоснование исследования

2.1 Вступление

Дегенеративная болезнь пояснично-крестцового отдела позвоночника является одним из наиболее распространенных хронических заболеваний. Общая встречаемость поясничного стеноза позвоночного канала, сопровождающегося ухудшением качества жизни, в России составляет около 5% среди пациентов до 50 лет и около 10—15% среди больных в возрасте 50—70 лет. Так же данная патология является одной из наиболее частых причин декомпрессивно-стабилизирующих вмешательств на поясничном отделе позвоночника у пациентов старше 50 лет [1]. Хирургическое лечение, как правило, заключается в ликвидации костных и связочных структур, компримирующих корешки конского хвоста. Вследствие резекции существенной части фасеточных суставов может развиться нестабильность соответствующего сегмента позвоночника или кифотическая деформация, в связи с чем необходимо выполнить межтеловой спондилодез при помощи кейджа и транспедикулярной фиксирующей системы.

Одним из наиболее спорных и нерешенных вопросов в хирургии дегенеративных заболеваний позвоночника является необходимость проведения стабилизации позвоночно-двигательного сегмента после декомпрессии у пациентов со степенями стеноза C и D по Shizas [2]. Опубликованный в 2020 году мета-анализ демонстрирует противоречивые данные [3]. С одной стороны, авторы приходят к тому, что клинически значимой разницы между двумя вышеуказанными методами вмешательство нет. С другой стороны, они основаны на ретроспективных исследованиях, типичным недостатком которых была неоднородность исследуемых выборок больных. Как

правило, более тяжелым группам пациентов с тяжелыми сопутствующими заболеваниями или с рисками псевдоартроза выполняли менее инвазивные методы операций, избегая стабилизации позвоночника.

Сетевой мета-анализ [4] продемонстрировал результаты сравнения 20 проспективных рандомизированных исследований различных методов лечения дегенеративных заболеваний позвоночника, из которых только в одном авторы попытались решить изучаемую нами проблему [5]. Данное исследование было инициировано 15 лет назад и имело ряд существенных недостатков, не позволяющих получить конкретный ответ на вопрос о преимуществах одной из используемых тактик лечения пациентов. Самыми существенными являются высокая степень гетерогенности пациентов в отношении степени и протяженности стенозов и многообразие методик выполненных хирургических вмешательств. Выбор метода декомпрессии стоял целиком за оперирующим хирургом. В тоже время, минимально инвазивная ламинотомия может демонстрировать более лучшие клинические результаты по сравнению с ламинэктомией за счет практически полного сохранения опорного аппарата позвоночно-двигательного сегмента. Авторами не был освящен вопрос восстановительного лечения в послеоперационном периоде, что также могло существенно повлиять на отдаленные клинические результаты и, соответственно, на финальный результат всего исследования. Также авторы не брали во внимание нарушения оси позвоночного столба, что также могло оказать существенное влияние на результаты лечения. Ни в одной работе, в том числе ретроспективной, мы не обнаружили сведений о возможных долгосрочных экономических преимуществах или недостатках различных методов

хирургического лечения одноуровневых дегенеративных стенозов позвоночника.

2.2 Цель исследования

Сравнить отдаленные клинические результаты применения двух методов хирургического лечения (декомпрессия позвоночного канала с или без проведения спондилодеза) у пациентов с одноуровневым стенозом позвоночного канала на поясничном уровне.

3 Оценка клинических исходов

3.1 Оценка первичного клинического исхода

Наибольшее значение в предстоящем исследовании будет иметь оценка клинического исхода в соответствии со шкалой Oswestry (ODI) [8]. Данный опросник позволяет точно в баллах оценить степень нарушения жизнедеятельности человека из-за болей в спине и объективно продемонстрировать клинический результат проведенного лечения. Шкала будет применена на всех этапах исследования, начиная с первичного осмотра, заканчивая контрольным осмотром через 2 года после вмешательства. Анкета включает в себя 10 разделов, содержащих по 6 утверждений. В зависимости от ответа присваивается балл (от 0 до 5). Максимальным количеством баллов является 50. После этого баллы переводят в проценты (от 0 до 100%). Если один из разделов неприменим или пропущен по этическим соображениям, то для расчёта процентов сумма баллов 9 разделов делится на 45.

3.2 Оценка вторичных клинических исходов

Наиболее всесторонне оценить качество жизни до и после проведенного вмешательства позволит шкала SF-36 v.1 (стандартная форма) [9]. Данный опросник позволяет провести всестороннюю оценку качества жизни. Анкета содержит 36 вопросов, которые сгруппированы в 8 шкал, оценивающих физическое функционирование, ролевую деятельность, телесную боль, общее здоровье, жизнеспособность, социальное функционирование, эмоциональное состояние и психическое здоровье. Для каждой из них максимальной оценкой является 100 баллов.

Вторая шкала оценки качества жизни будет EQ-5D-5L [10]. Данная шкала аналогична SF-36, однако, менее подробно оценивает социальную адаптацию пациентов и эмоциональную составляющую. Тем не менее, данный опросник очень часто применяют в проспективных международных исследованиях, посвященных лечению стенозов поясничного отдела позвоночника. В связи с этим мы считаем целесообразным его использование для сохранения возможности сравнительного анализа наших результатов с литературными данными. Анкета содержит 5 разделов (подвижность, уход за собой, обычная деятельность, боль, тревога) по три утверждения и визуально-аналоговую шкалу боли EQ-VAS. Результаты могут быть конвертированы в единое числовое значение (индекс) с поправкой на регион проживания пациента. В настоящее время необходимого набора значений для расчета данного индекса для РФ нет [11], поэтому на начальных этапах будет применено простое сравнение конкретных числовых значений для каждого параметра. При появлении технической возможности, индекс будет рассчитан в конце исследования.

Шкала оценки хронического болевого синдрома Вон-Корффа (chronic pain grade questionnaire, CPGQ) [12]. Позволяет в баллах оценить выраженность боли и ее влияние на жизнедеятельность. Степень 0 соответствует отсутствию боли, а степень IV – максимальное ограничение жизнедеятельности за счет выраженного болевого синдрома.

Шкала катастрофизации боли (The Pain Catastrophizing Scale (PCS) [13] позволяет полноценно оценить психологические аспекты восприятия боли, в особенности преувеличенное негативное восприятие болезненных стимулов. Опросник содержит 13 вопросов, каждый из которых оценивают в баллах (0

баллов – отсутствие симптома, 4 – пациент испытывает эти чувства все время).

Опросник совладания с болью Chronic Pain Coping Inventory [14] состоит из 64 вопросов, позволяющих отнести предпочитаемую пациентом стратегию преодоления хронической боли к одной из восьми шкал.

Перед операцией однократно будет проведена оценка физического статуса пациента по шкале американского общества анестезиологов ASA. Данная классификация выделяет 5 классов физического статуса в зависимости от наличия сопутствующих заболеваний (I класс – здоровый пациент, V класс – умирающий).

Также будет подсчитана стоимость госпитализации пациента, включая выполнение хирургического вмешательства, а также сроки нетрудоспособности пациента в связи с заболеванием позвоночника. Также, в течении 2 лет после вмешательства будут учтены в соответствии с МЭС стоимость последующего лечения в реабилитационных центрах и других стационарах по поводу осложнений операции или других проявлений дегенеративно-дистрофических изменений поясничного отдела позвоночника.

Для всех пациентов после инструментальной фиксации будет проведена оценка качества спондилодеза в соответствии с критериями Tan et al. [15]. Степень 1 соответствует полноценному костному сращению, а степень 4 – псевдоартрозу. Помимо этого, также будет зафиксирован срок формирования спондилодеза.

Для всех пациентов будут проанализированы параметры сагиттального баланса: наклон таза (PI), отклонение таза от вертикали (PT), наклон S1 позвонка (SS), смещение вертикальной оси (SVA).

4 УЧАСТНИКИ ИССЛЕДОВАНИЯ

Это исследование будет проводиться с участием научного и клинического персонала всех больниц.

Главный исследователь: проф. А.А. Гринь, д.м.н., член-корр. Академии РАН, руководитель отделения неотложной нейрохирургии ГБУЗ НИИ СП им. Н.В. Склифосовского ДЗМ, GrinAA@sklif.mos.ru

Исследовательский коллектив: Талыпов А.Э., д.м.н., в.н.с. отделения неотложной нейрохирургии ГБУЗ НИИ СП им. Н.В. Склифосовского ДЗМ, TalypovAE@sklif.mos.ru

Львов И.С., к.м.н., с.н.с. отделения неотложной нейрохирургии ГБУЗ НИИ СП им. Н.В. Склифосовского ДЗМ, LvovIS@sklif.mos.ru

Кордонский А.Ю., к.м.н., с.н.с. отделения неотложной нейрохирургии ГБУЗ НИИ СП им. Н.В. Склифосовского ДЗМ, KordonskiyAJ@sklif.mos.ru

Смирнов В.А., к.м.н., с.н.с. отделения неотложной нейрохирургии ГБУЗ НИИ СП им. Н.В. Склифосовского ДЗМ, SmirnovVV@sklif.mos.ru

Лебедев В.Б., к.м.н., врач-травматолог отделения нейрохирургии ФГБУ «НМХЦ им. Н.И. Пирогова» Минздрава России, horizont_vbl@mail.ru

Ответственные исполнители: Зуев С.Е., н.с. отделения неотложной нейрохирургии ГБУЗ НИИ СП им. Н.В. Склифосовского ДЗМ, ZuevSE@sklif.mos.ru, +7 (915) 377-...-...

Сосновский Е.А., к.м.н., врач-нейрохирург отделения неотложной нейрохирургии ГБУЗ НИИ СП им. Н.В. Склифосовского ДЗМ, SosnovsliyEA@sklif.mos.ru, +7(926) 572-...-...

Епифанов Д.С., врач-нейрохирург отделения нейрохирургии ФГБУ «НМХЦ им. Н.И. Пирогова» Минздрава России, +7 (965) 302-...-...

Календари А.А., д.м.н., в.н.с. отделения неотложной нейрохирургии ГБУЗ НИИ СП им. Н.В. Склифосовского ДЗМ, +7 (925) 484-...-...

5 Дизайн исследования и его регистрация

Это исследование является открытым, многоцентровым, рандомизированным контролируемым исследованием. Все зарегистрированные субъекты будут разделены на две параллельные группы в зависимости от применяемой хирургической техники (декомпрессия или декомпрессия со слиянием).

Настоящий протокол написан в соответствии с рекомендациями SPIRIT (Standard Protocol Items: Recommendations for Interventional Trials – стандартный рекомендательный протокол для интервенционных исследований) [6].

Исследование зарегистрировано в базе данных http://www.clinicaltrials.gov (номер NCT05273879). Результаты исследования будут отражены в соответствии с общепринятыми стандартами CONSORT (Consolidated Standards of Reporting Trials) [7].

6 Отбор пациентов и описание операций

6.1 Участники исследования

Всего в исследовании планируется участие хирургов из 3 стационаров, оказывающих плановую хирургическую помощь пациентам с дегенеративно-дистрофическими заболеваниями позвоночника: 1. ГБУЗ НИИ СП им. Н.В. Склифосовского ДЗМ; 2. ФГБУ «НМХЦ им. Н.И. Пирогова» Минздрава России; 3. ФГБУ «Федеральный центр мозга и нейротехнологий» ФМБА.

Все участники имеют большой опыт (более 12 лет) в хирургии дегенеративных заболеваний позвоночника и проводят не менее 180 операций в год. Обе хирургические методики (декомпрессия и декомпрессия со спондилодезом) используются во всех трех клинических центрах уже более 10 лет. Ранее в рутинной практике спондилодез обычно применяли в случаях нестабильности, подтвержденной рентгенологически или интраоперационно. Методика декомпрессии выбиралась на основании личного мнения хирурга. Тем не менее, все хирурги имеют большой опыт в обоих типах операций.

Восстановительное лечение всем пациентам будет проведено по стандартной программе на базе филиала №3 ГАУЗ «Московский научно-практический центр медицинской реабилитации, восстановительной и спортивной медицины Департамента здравоохранения города Москвы». В исследовании примут участие три опытных реабилитолога. Чтобы избежать гетерогенности послеоперационного лечения, они будут использовать единый протокол для послеоперационного лечения всех пациентов в исследовании.

6.2 Отбор пациентов

Первичный отбор пациентов будет осуществлен на догоспитальном этапе. В исследование будут включены пациенты возрастом от 45 до 75 лет с симптомным одноуровневым стенозом на уровнях L2-L3, L3-L4, L4-L5 или L5-S1. Критерии включения и исключения из исследования представлены в таблице 1. Если пациенты, подходящие по критериям включения, не будут продолжать участие в исследовании, данный факт будет зарегистрирован с соответствующим пояснением.

Все пациенты будут проинформированы о предстоящем исследовании. Письменное и устное согласие пациентов на участие будут обязательны перед процедурой рандомизации. Процедура отбора пациентов и их участие в исследовании отражены на рис. 1.

Таблица 1

Критерии включения и исключения пациентов из исследования

Критерии включения:<br>- возраст пациентов от 45 до 75 лет;<br>- степень стеноза C или D в соответствии с Shizas et al. по данным МРТ на уровне L2-L3, L3-L4, L4-L5 или L5-S1;<br>- клинические проявления поясничного стеноза (синдром нейрогенной клаудикации и/или радикулопатия);<br>- отсутствие эффекта от консервативной терапии в течение 3 месяцев;<br>- информированное согласие на участие в исследовании.
Критерии невключения:<br>- спондилолистез позвонка более 3мм;<br>- нестабильность сегмента по данным функциональной рентгенографии;<br>- сагиттальный дисбаланс (тип 4 по C.Barrey);<br>- плотность костной ткани позвонков на уровне операции менее 100 HU.<br>- клинически значимый стеноз позвоночника на 2 и более уровнях;<br>- ранее выполненные операции на позвоночнике;<br>- риск наркоза 4 или 5 по ASA;<br>- невозможность участвовать в контрольных осмотрах в течении 2 лет после операции;<br>- участие в других клинических исследованиях, связанных с хирургическим или консервативным лечением заболеваний позвоночника.
Критерии исключения из исследования:<br>- отзыв информированного согласия;<br>- пропуск пациентом запланированных контрольных осмотров;<br>- развитие осложнений или других заболеваний, требующих смены выбранной тактики лечения.

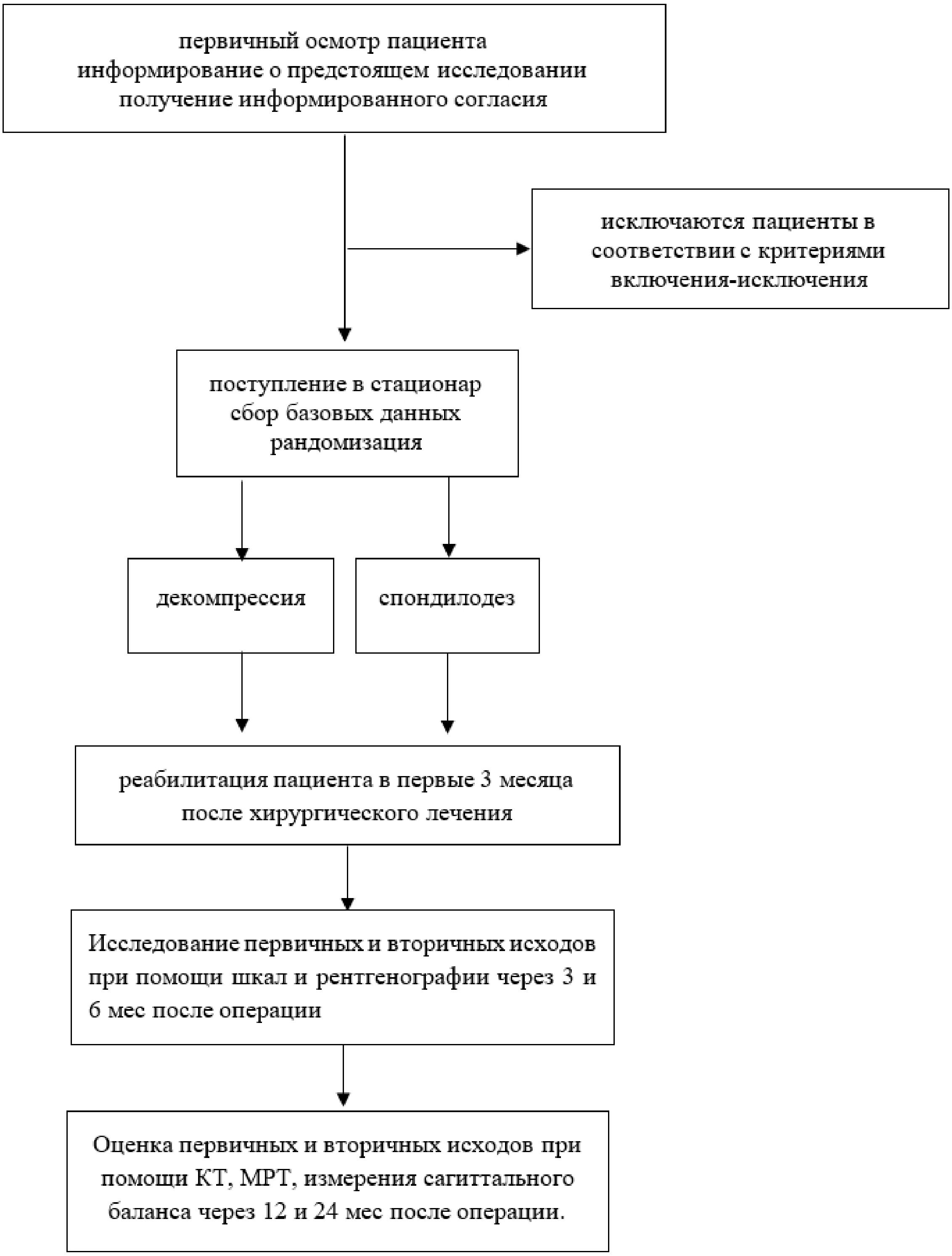

Рис. 1 Основные этапы участия пациента в исследовании

6.3 Методы хирургического лечения

Все хирургические процедуры будут проведены под эндотрахеальным наркозом.

В группе декомпрессии позвоночного канала сначала, с одной стороны, будет выполнена ламинотомия соответствующих смежных позвонков, парциальная флавэктомия и медиальная фасетэктомия. Далее в зависимости от личных предпочтений хирурга возможно 2 варианта операции: 1) аналогичная процедура декомпрессии с другой стороны; 2) контрлатеральная декомпрессия. Независимо от выбранного варианта, у всех пациентов данной группы должны быть сохранены в целостности остистый отросток, межостистые и надостистые связки, часть фасеточных суставов и соответствующей дужки позвонка.

В группе декомпрессии и стабилизации алгоритм операции следующий. Сначала выполняют декомпрессию по одной из вышеописанных методик. Далее выполняют трансфораминальный межтеловой спондилодез кейджем и фиксацию транспедикулярными винтами.

6.4 Процедура рандомизации

Пациенты, соответствующие критериям включения и подписавшие согласие на участие в исследовании, случайным образом в соотношении 1:1 будут разделены на 2 группы в зависимости от метода хирургического вмешательства. Будет использована блочная рандомизация со стратификацией по степени стеноза (C и D по Shizas). Выбор метода лечения будет проведен за сутки перед хирургическим вмешательством специалистом, не вовлеченным в осмотр и лечение участвующих пациентов. Результаты рандомизации будут задокументированы в истории болезни. Исследование предполагается открытым,

соответственно пациент будет информирован о виде выбранного хирургического вмешательства, однако, ни больной, ни хирург повлиять на выбор метода операции не смогут.

6.5 Ослепление

Планируется, что это будет открытое исследование. Все зарегистрированные участники будут проинформированы о выбранном типе операции. Важно отметить, что в каждом случае ни пациент, ни хирург не будут иметь никакого влияния на выбор хирургической техники. Последующие оценки результатов (таблица 2) будут проводиться клиницистами, не участвующими в лечении пациента.

7 Сбор данных

Координаторы исследования во всех клинических центрах несут ответственность за сбор и администрирование данных на всех этапах исследования. Все обследования (анкеты, опросники, рентгенография и КТ) будут проводиться во время личных контрольных посещений в учреждении, где было выполнено хирургическое лечение. Все полученные данные будут храниться в НИИ СП им. Н.В. Склифосовского. Эти данные будут недоступны для всех сторон до момента проведения окончательного анализа. График сбора данных представлен в таблице 2.

Таблица 2

График сбора данных в исследовании

	Перед госпитализацией	Госпитализация	3 мес п/о	6 мес п/о	12 мес п/о	24 мес п/о
Демографические и клинические данные	+					
Информированное согласие	+					
Клинические шкалы						
ODI	+		+	+	+	+
SF-36	+		+	+	+	+
EQ-5D-5L	+		+	+	+	+
ASA	+					
Психологические шкалы						
CPGQ	+		+	+	+	+
PCS	+		+	+	+	+
CPCI	+		+	+	+	+
Рентгенография поясничного отдела позвоночника		+	+	+		
Измерение сагиттального баланса	+				+	+
КТ	+				+	+
МРТ	+				+	+
Рандомизация		+				
Операция		+				
Стоимость лечения		+				
Стоимость операции			+			
Стоимость лечения в других учреждениях			+	+	+	+
Осложнения, реоперации		+	+	+	+	+
Контрольные осмотры			+	+	+	+

8 Статистика

8.1 Расчет выборки пациентов

В соответствии с non-inferiority дизайном мы рассчитали размер выборки на основании первичного исхода. Согласно последнему рандомизированному исследованию [5], стандартное отклонение ODI в группе спондилодеза составляет 20 баллов. При границе не меньшей эффективности $\delta = 12$, двустороннем уровне α 0,05, мощности 80% и соотношении распределения 1:1 требуемый размер выборки составляет 70 пациентов. Учитывая вероятность того, что 20% пациентов пропустят обязательные последующие осмотры, размер выборки составит 86 пациентов (по 43 пациента в группу).

8.2 Статистический анализ

Нормальность переменных будет оцениваться с использованием критерия Шапиро-Уилка. Критерий Стьюдента (для данных с нормальным распределением) и критерий хи-квадрат Пирсона (для категориальных данных) будут использоваться для выявления различий в частоте симптомов среди групп. Множественная линейная регрессия будет использоваться для измерения точечных оценок и доверительных интервалов для групповых различий в показателях ODI и клинических шкал от исходного уровня до 2-летнего наблюдения. Статистическая значимость будет определяться как $p < 0,05$ на основе двустороннего теста.

Непараметрический U-критерий Манна-Уитни будет использоваться для выявления различий в непрерывных переменных между группами как до, так и после операции. Непрерывные данные внутри групп будут сравниваться с

использованием теста Уилкоксона. Категориальные параметры среди групп будут сравниваться с использованием точного двустороннего критерия Фишера.

Частота осложнений в течение 2 лет наблюдения будет сравниваться между группами с использованием кривых выживаемости Каплана-Мейера и логарифмического рангового теста. Значимость факторов риска для всех зарегистрированных осложнений будет оцениваться с использованием логистической регрессии.

Для отсутствующих данных о результатах будет использоваться метод множественного восстановления.

9 Промежуточный анализ и прекращение исследования

Промежуточный анализ будет проведен, когда будут доступны данные контрольного обследования через 12 месяцев как минимум для 20 пациентов каждой группы. Критериями остановки исследования являются: 1) если количество реопераций в одной из групп будет превышать 50%; 2) если в одной из групп будет выше частота отрицательной динамики в неврологическом статусе после проведенного лечения со снижением ODI ниже исходного уровня на 10 баллов и более; 3) если количество хирургических осложнений в одной из групп будет превышать 50%.

Промежуточный анализ будет проведен исследователем, не участвующим в лечении пациентов. Статисту будут доступны данные неврологического осмотра и ODI на всех этапах исследования, а также известна частота реопераций для представленных групп пациентов.

10 Этические аспекты исследования

Исследование будет проведено в соответствии с Хельсинкской декларацией Всемирной Медицинской Ассоциации. Протокол данного исследования был одобрен этическими комитетами каждого из участвующих учреждений (НИИ СП им. Н.В. Склифосовского, №1-22/ 11.01.22; НМХЦ им. Н.И. Пирогова, №2/16.02.2022; Федеральный центр мозга и нейротехнологий, №01 / 04-03-22).

Все данные по предстоящему исследованию будут храниться на базе НИИ СП им. Н.В. Склифосовского. Протокол, предварительные и окончательные результаты исследования будут представлены в рецензируемых журналах, посвященных нейрохирургии и спинальной хирургии, а также соответствующих конференциях.

11 Финансирование

Исследование будет профинансировано за счет бюджетов участвующих стационаров. Сборы за публикации статей будут оплачены Московским Центром Инновационных Технологий в Здравоохранении.

12 Список литературы

1. Otani K., Kikuchi S., Yabuki S. et al. Lumbar spinal stenosis has a negative impact on quality of life compared with other comorbidities: an epidemiological cross-sectional study of 1862 community dwelling individuals. Scientific World Journal 2013;2013:590652. DOI: 10.1155/2013/590652.
2. Schizas C, Theumann N, Burn A, Tansey R, Wardlaw D, Smith FW, et al. Qualitative grading of severity of lumbar spinal stenosis based on the morphology of the dural sac on magnetic resonance images. Spine. 2010;35: 1919–1924. 10.1097/BRS.0b013e3181d359bd
3. Ma H, Hai B, Yan M, Liu X, Zhu B. Evaluation of Effectiveness of Treatment Strategies for Degenerative Lumbar Spinal Stenosis: A Systematic Review and Network Meta-Analysis of Clinical Studies. World Neurosurg. 2021 Aug;152:95-106. doi: 10.1016/j.wneu.2021.06.016.
4. Wei FL, Zhou CP, Liu R, Zhu KL, Du MR, Gao HR, Wu SD, Sun LL, Yan XD, Liu Y, Qian JX. Management for lumbar spinal stenosis: A network meta-analysis and systematic review. Int J Surg. 2021 Jan;85:19-28. doi: 10.1016/j.ijsu.2020.11.014. Epub 2020 Nov 27. PMID: 33253898.
5. Försth P, Ólafsson G, Carlsson T, Frost A, Borgström F, Fritzell P, Öhagen P, Michaëlsson K, Sandén B. A Randomized, Controlled Trial of Fusion Surgery for Lumbar Spinal Stenosis. N Engl J Med. 2016 Apr 14;374(15):1413-23. doi: 10.1056/NEJMoa1513721. PMID: 27074066.
6. Agha RA, Altman DG, Rosin D. The SPIRIT 2013 statement--defining standard protocol items for trials. Int J Surg. 2015 Jan;13:288-291. doi: 10.1016/j.ijsu.2014.12.007.
7. Piaggio G, Elbourne DR, Altman DG, Pocock SJ, Evans SJ; CONSORT Group. Reporting of noninferiority and equivalence randomized trials: an extension of the CONSORT statement. JAMA. 2006 Mar 8;295(10):1152-60. doi: 10.1001/jama.295.10.1152.

8. Bakhtadze M.A., Bolotov D.A., Kuzminov K.O. Oswestry Disability Index: A Study of Relibility and Validity of the Russian Version. Manual therapy (in Russ) 2016; 4(64): 24-33.

9. Amirdjanova V.N., Goryachev D.V., Korshunov N.I., Rebrov A.P., Sorotskaya V.N. SF-36 questionnaire population quality of life indices Objective. Rheumatology Science and Practice. 2008;46(1):36-48. (In Russ.) https://doi.org/10.14412/1995-4484-2008-852

10. Akulova A.I., Gaydukova I.Z., Rebrov A.P. VALIDATION OF THE EQ-5D-5L VERSION IN RUSSIA. Rheumatology Science and Practice. 2018;56(3):351-355. (In Russ.) https://doi.org/10.14412/1995-4484-2018-351-355

11. https://euroqol.org/

12. Von Korff M, Ormel J, Keefe FJ, Dworkin SF. Grading the severity of chronic pain. Pain. 1992 Aug;50(2):133-149. doi: 10.1016/0304-3959(92)90154-4. PMID: 1408309.

13. Radchikova N.P., Adashiskaya G.A., Sanoyan T.R., Shupta A.A. Russian Adaptation of the Pain Catastrophizing Scale. Clinical Psychology and Special Education 2020; 9(4): 169–187. DOI: 10.17759/cpse.2020090409

14. Jensen MP, Turner JA, Romano JM, Strom SE. The Chronic Pain Coping Inventory: development and preliminary validation. Pain. 1995 Feb;60(2):203-216. doi: 10.1016/0304-3959(94)00118-X. PMID: 7784106.

15. Tan GH, Goss BG, Thorpe PJ, et al. Ct-Based classification of long spinal allograft fusion. Eur Spine J 2007;16:1875–81.

Приложение 1. Информация для пациента и согласие на участие в исследовании

Часть 1. Информационный блок

Вступление

Уважаемый пациент! Мы благодарим Вас за проявленный интерес к нашему исследованию. У Вас был установлен диагноз «остеохондроз позвоночника, дегенеративный полифакторный стеноз позвоночного канала», требующий проведения хирургического вмешательства. Во вступительной части информационного блока мы хотели бы проинформировать Вас об основных аспектах данного заболевания и возможных вариантах лечения.

Дегенеративная болезнь пояснично-крестцового отдела позвоночника является одним из наиболее распространенных хронических заболеваний. Общая встречаемость поясничного стеноза позвоночного канала, сопровождающегося ухудшением качества жизни, в России составляет около 5% среди пациентов до 50 лет и около 10—15% в возрастной группе 50—70 лет. В абсолютном большинстве случаев данная патология является приобретенной и обусловлена прогрессированием остеохондроза позвоночника (артроз, увеличение в объеме суставов позвоночника, утолщение связок, формирование костных разрастаний – «остеофитов» - на телах позвонков). Позвоночник помимо важной опорной функции выполняет роль вместилища для спинного мозга и спинномозговых нервов, обеспечивающих проведение сигнальных импульсов от головного мозга к соответствующим органам и системам. Вследствие вышеперечисленных процессов все структуры позвоночника увеличиваются в размерах, из-за чего позвоночный канал сужается, а проходящие в нем спинной мозг и нервные корешки подвергаются сдавлению (рис. 1).

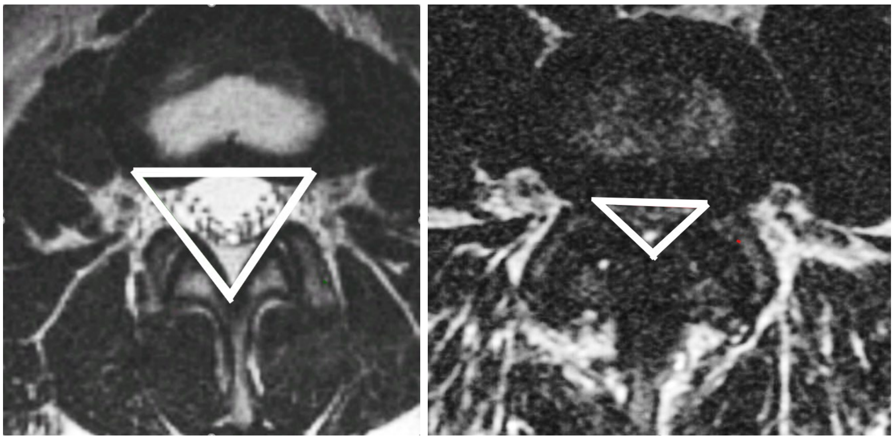

а б

Рис. 1 Пример стеноза просвета позвоночного канала по данным МРТ. Позвоночный канал обозначен на рисунках треугольниками: а – позвоночный канал нормальных размеров; б – позвоночный канал сужен за счет стеноза.

Клинические проявления при стенозе поясничного отдела позвоночника обусловлены как непосредственным сдавлением нервов, проходящих в позвоночном канале, так и сдавлением прилежащих сосудов. Снижение кровотока ко всему «пучку» нервов на уровне поражения со временем приводит к необратимым изменениям с

нарушением двигательной функции, вплоть до глубокой инвалидизации пациента. Соответственно, основными клиническими проявлениями являются:

1) боль в спине;

2) синдром перемежающейся нейрогенной хромоты (усиливающаяся боль при ходьбе на небольшие расстояния, уменьшающаяся при присаживании или наклоне вперед);

3) боль в ноге по ходу сдавленного нервного корешка;

4) слабость, атрофия соответствующих мышц ног.

Диагноз «поясничный стеноз» может быть установлен на основании комплекса клинических данных и результатов магнитно-резонансной томографии (МРТ). Прочие исследования (компьютерная томография, рентгенография, электронейромиография) носят вспомогательный характер и используются для всестороннего уточнения возникших в позвоночнике изменений и предоперационного планирования.

В настоящее время наиболее распространено деление стенозов на 4 степени (A, B, C, D) на основании данных МРТ. Степень A является наиболее легкой, а степень D соответствует практически полному «зарастанию» канала позвоночника с грубым сдавлением всех проходящих в нем структур.

Во всех случаях симптомного поясничного стеноза начальным методом лечения является консервативная терапия. Под контролем невролога по месту жительства пациент получает курс противовоспалительной, противоотечной, сосудистой терапии, проходит курс физиотерапии и лечебной физкультуры. Курс консервативной терапии должен продолжаться не менее 3 месяцев. Если улучшения у пациента не наступило, то возможно, речь идет о декомпенсации заболевания и необходимо обращение к нейрохирургу для хирургического лечения. Необходимо иметь ввиду, что *любая* степень стеноза может быть полностью асимптомной и тогда она не требует хирургического лечения!

Основной целью хирургического лечения является расширение позвоночного канала путем удаления «разросшихся» костных и связочных структур, сдавливающих нервно-сосудистые образования. При некоторых методиках операций, широкая декомпрессия приводит к ликвидации важных опорных структур позвоночника, что может послужить причиной смещения позвонков и усугубления клинической картины (нестабильность позвоночника). Следует отметить, что современные методы декомпрессии с применением микроскопа и микрохирургического инструментария позволяют существенно снизить необходимый объем резекции задних структур позвоночника и уменьшить вероятность развития нестабильности.

Часто, для профилактики развития данной нестабильности хирург выполняет фиксацию позвоночника с целью создания спондилодеза («сращения» позвонков на уровне операции). Для этого полностью удаляют межпозвоночный диск, вместо него устанавливают протез, а сами позвонки фиксируют специальной системой винтов с стержней.

Один из наиболее важных вопросов современной хирургии поясничных стенозов в настоящее время следующий: «нужно ли фиксировать металлоконструкцией сегмент позвоночника после декомпрессии позвоночного канала, если сохранены важные опорные структуры позвоночника?». К настоящему времени, только одно исследование с *высоким уровнем доказательности* было проведено в попытке решения этой проблемы

(Försth P et al. A Randomized, Controlled Trial of Fusion Surgery for Lumbar Spinal Stenosis. N Engl J Med. 2016 Apr 14;374(15):1413-23). К сожалению, оно неоднократно было подвергнуто существенной критике из-за большого количества недостатков в наборе групп пациентов и выбора методов хирургического вмешательства. Из-за этого, результаты данного исследования не могут быть рассмотрены в современной медицине в качестве стандартного решения.

Все остальные опубликованные исследования имеют *низкий уровень доказательности* и зачастую сводятся к личному опыту конкретной клиники в применении того или иного метода лечения и в каждом из них были получены совершенно различные противоположные результаты. Если обобщить эти данные, то можно выделить следующие выводы относительно применения металлофиксаторов при таких операциях:

1. Улучшение клинической картины более значительное при стабилизации позвоночника за счет возможности более широкой резекции костных структур;
2. Фиксация позвоночника после выполнения декомпрессии при стенозах степени C и D снижает частоту реопераций на данном конкретном сегменте позвоночника в ближайшие 10 лет после операции;
3. Фиксация позвоночника после выполнения декомпрессии при стенозах степени C и D снижает вероятность деформации позвоночника после хирургического вмешательства.

В то же время существуют и отрицательные аспекты применения систем фиксации позвоночника:

1. Фиксация позвоночника после выполнения декомпрессии при стенозах степени C и D увеличивает интраоперационную кровопотерю и частоту ранних операционных осложнений, продолжительность госпитализации;
2. Фиксация позвоночника *достоверно* увеличивает длительность операции;
3. Применение имплантов увеличивает вероятность формирования синдрома смежного уровня с провоцированием развития стеноза позвоночного канала выше и ниже уровня операции;
4. Сам факт пожизненного присутствия титановых имплантов в позвоночнике.

При этом, с применением современных минимально инвазивных технологий при стенозах степени C и D может быть достигнута достаточная декомпрессия корешков без широкой резекции костных структур (ламинэктомии).

Описание исследования

Почему необходимо ознакомиться с данной информацией?

Вы были приглашены для участия в исследовании, потому что у Вас был диагностирован стеноз позвоночного канала на поясничном уровне со степенью выраженности C или D. Перед тем, как Вы примете участие в исследовании, необходимо ознакомиться с целью данной работы, предстоящими процедурами, возможными рисками и неудобствами, связанными с участием в исследовании. Если при ознакомлении с настоящим материалом возникнут какие-либо вопросы, на них сможет ответить осматривающий Вас врач, ответственные исполнители или руководитель исследования, координаты которых будут указаны на отдельной странице. Таким образом, Вы сможете принять максимально информированное согласие для участия в исследовании.

Цель исследования

Главной целью исследования является сравнение эффективности применения двух методов хирургического лечения у пациентов с одноуровневым стенозом позвоночного канала на поясничном уровне. В исследовании не будут применяться новые

экспериментальные методы лечения. Оба варианта операции являются рутинными, отработанными методиками, официально разрешенными к применению в РФ соответствующими компетентными структурами. У одной группы пациентов будет выполнена только декомпрессия нервно-сосудистых образований позвоночного канала, у другой точно такая же декомпрессия будет дополнена стабилизацией позвоночника. Преимущества и недостатки обоих методов были описаны во вступительной части информационного блока. Основной вопрос, который будет решен в исследовании – выбор наиболее эффективного метода лечения.

Продолжительность исследования

Участие в исследовании займет 2 года. Примут участие 86 пациентов, проживающих на территории РФ и имеющие возможность своевременно являться на контрольные осмотры. Все лечебные и диагностические процедуры, проведенные в ходе настоящего исследования, будут оплачены за счет фонда ОМС, ВМП или бюджета учреждения, участвующего в исследовании.

Обязанности пациента во время исследования

Во время исследования мы попросим участников исследования выполнять ряд требований, направленных на своевременное мониторирование состояние их здоровья, а именно:

- полностью сообщить анамнестические данные касательно основного и сопутствующих заболеваний, а также препараты, которые пациент принимал ранее или продолжает принимать на момент участия в исследовании;

- не пропускать запланированные визиты на контрольные осмотры;

- своевременно сообщать об изменении своего состояния, каких-либо жалобах или симптомах, а также визитах в поликлинику или другие стационары для получения медицинской помощи;

- отказаться от участия в настоящем исследовании, если существует высокая вероятность пропуска контрольных осмотров или при участии в иных клинических испытаниях.

Последовательность мероприятий и процедур в ходе исследования

Первичный осмотр

Во время первичного осмотра на догоспитальном этапе Вы будете осмотрены компетентным специалистом, который оценит возможность участия в исследовании на основании определенных критериев включения и исключения. После разъяснения сути предстоящей работы, он получит Ваше письменное согласие на участие и соберет все необходимые данные для госпитализации в стационар.

Госпитализация и выбор метода лечения

В первый день госпитализации лечащим врачом у Вас буду собраны необходимые базовые данные, включающие в себя демографические показатели, информацию о сопутствующих заболеваниях, историю Вашего заболевания. Также необходимо будет заполнить следующие анкеты: Oswestry Disability Index (ODI), шкала оценки качества жизни SF-36, опросник EuroQol Five-Dimensional (EQ-5D), шкала оценки хронического болевого синдрома Вон Корф (chronic pain grade questionnaire, CPGQ), шкала катастрофизации боли

(The Pain Catastrophizing Scale (PCS), опросник совладания с болью Chronic Pain Coping Inventor. Дополнительно Вы будете осмотрены неврологом, медицинским психологом и анестезиологом. Окончательный выбор метода лечения будет осуществлен за сутки до предстоящего вмешательства на консилиуме оперирующего хирурга и исследовательской группы. Они просмотрят анамнестические и клинические данные, необходимые медицинские записи о состоянии здоровья, снимки МРТ, КТ и Rg. Если пациент будет полностью соответствовать критериям включения и не будет иметь противопоказаний, то ему при помощи метода блочной рандомизации будет выбран метод хирургического лечения. Результаты консилиума будут отражены в истории болезни.

Хирургическое лечение

Все операции будут выполняться хирургом с опытом спинальных операций не менее 10 лет, выполняющим ежегодно не менее 100 вмешательств на позвоночнике. В зависимости от заключения консилиума, будет выполнен один из вариантов хирургического вмешательства:

1. микрохирургическая декомпрессия нервных структур позвоночного канала с сохранением в целостности заднего опорного комплекса позвоночника.

2. микрохирургическая декомпрессия нервных структур позвоночного канала дополненная комбинированным спондилодезем кейджем и транспедикулярными винтами по технологии TLIF (трансфораминальный межтеловой поясничный спондилодез).

Риски хирургического лечения

Во время госпитализации в стационар Вам также необходимо будет подписать стандартное согласие на хирургическое вмешательство. Лечение, в том числе хирургическое, может нести потенциальные риски для здоровья. В любом случае перед операцией Вы будете проинформированы о всех возможных рисках хирургического лечения. Участие в настоящем исследовании не подразумевает использование экспериментальных методов или малоизученных технологий. Все применяемые методы операций являются отработанными методиками, которые применяются в РФ более 15 лет.

Выписка из стационара

Перед выпиской из стационара Вам будет выполнена контрольная КТ поясничного отдела позвоночника для оценки точности установленных имплантов и качества декомпрессии. Будет выполнен контрольный осмотр неврологом с оценкой динамики радикулярного синдрома. Также с Вами будет составлен график контрольных осмотров через 3, 6, 12 и 24 мес.

Этап восстановительного лечения

Все пациенты после операции должны будут пройти курс восстановительного лечения на базе реабилитационного центра ГАУЗ МНПЦ МРВСМ ДЗМ. После выписки из реабилитационного центра будут даны необходимые рекомендации по дальнейшему образу жизни. Всем участникам исследования необходимо будет их придерживаться для профилактики прогрессирования клинических проявлений остеохондроза.

Осмотры через 3 и 6 мес.

Во время данных визитов Вы будете осмотрены нейрохирургом. Будут собраны следующие данные: наличие жалоб, общее состояние здоровья, прием каких-либо медикаментов, госпитализации в стационары или обращения в медицинские учреждения

по любым причинам. Вам необходимо будет заполнить специальные анкеты, позволяющие оценить качество жизни на момент осмотра и его изменения после последнего контрольного осмотра. На основании данных рентгенографии будет оценено положение имплантов и изменения углов деформации позвоночника.

Осмотры через 12 и 24 мес.

Помимо вышеперечисленных данных (осмотры специалистов и заполнение анкет), Вам необходимо будет пройти комплексное обследование, включающее в себя:

- контрольная КТ зоны операции для оценки степени костного сращения или имплант-ассоциированных осложнений (для стабилизирующей операции) или состояние диска и суставов оперированного сегмента (при декомпрессивном вмешательстве);

- МРТ поясничного отдела позвоночника для выявления изменений в области операции и смежных уровнях позвоночника;

- рентгенография всего позвоночника для оценки глобального баланса.

Окончание участия в исследовании

На контрольном осмотре через 24 месяца после операции лечащий врач, если это необходимо, даст Вам дальнейшие рекомендации касательно образа жизни и восстановительного лечения. В случае развития каких-либо осложнений или сохраняющегося болевого синдрома Вам будет предложено паллиативное лечение на базе стационара или Вы будете направлены в соответствующий специализированный центр.

Участие в исследовании добровольное. На любом этапе Вы можете прекратить свое участие без объяснения причин. В случае отказа, мы просим Вас своевременно предупредить врача, ответственного за проведение исследования или оперирующего хирурга. Обращаем внимание, что Вы не сможете участвовать в альтернативных исследованиях лечения дегенеративных заболеваний позвоночника до тех пор, пока не прекращено Ваше участие в данной работе.

Материальная компенсация за участие в исследовании

Денежная или иная форма компенсации за участие в исследовании не предусмотрены. Исследование организовано без привлечения спонсоров. Все траты, включая необходимые обследования и лечение будут проведены за счет средств ОМС, ВМП или за счет бюджета участвующих учреждений. В случае развития осложнений в результате операции, все лечение будет проведено бесплатно.

Исключение из проведения исследования

На любом этапе исследования Ваше участие может быть завершено по следующим причинам:

1. Необходимо дополнительное лечение, не включенное в настоящий протокол.

2. Вы не следуете инструкциям лечащего врача.

3. В ходе исследования развилось состояние, соответствующее критериям исключения.

4. Уже набрано необходимое число пациентов.

5. Исследование было остановлено одной из организаций или уполномоченными государственными органами.

Использование персональных данных

Участие в исследовании подразумевает сбор персональных данных. Соблюдение приватности и сохранность персональных данных является одной из приоритетных задач. Информация, позволяющая Вас идентифицировать, будет известна руководителю исследования и ответственным исполнителям. В доступных всем исследователям базах данных Вам будет присвоен порядковый номер. В отчетах и публикациях результатов исследования идентифицировать Вашу личность будет невозможно.

Подписывая согласие на участие в данном исследовании, Вы даете добровольное согласие на использование необходимых персональных данных, а именно: фамилия, имя, отчество, пол, возраст, адрес, контактный телефон, данные касательно образа жизни, история заболевания, вес, рост. Без данной подписи мы не имеем право использовать Ваши персональные данные в исследовании и поэтому Ваше участие в нем не будет возможным.

Вы имеете право в любой момент исследования исправить или удалить свои персональные данные. Также, Вы можете в любой момент отказаться от участия в исследовании, в том числе на этапе сбора базовой информации.

Информация о результатах исследования

Описание результатов исследования будет доступно на сайте http://www.clinicaltrials.gov/. Никакой персональной информации на данном сайте опубликовано не будет.

Контактная информация

Если у Вас возникнут какие-либо вопросы касательно исследования, Вы можете связаться с ответственными исполнителями и руководителем исследования, а также этическим комитетом одного из участвующих учреждений.

Главный исследователь: Гринь Андрей Анатольевич, д.м.н., заведующий научным отделом неотложной нейрохирургии НИИ СП им. Н.В. Склифосовского, профессор кафедры нейрохирургии и нейрореанимации МГМСУ им. А.И. Евдокимова, главный внештатный нейрохирург Департамента Здравоохранения Г. Москвы. GrinAA@sklif.mos.ru

Исследователи:

Талыпов Александр Эрнестович*, д.м.н., ведущий научный сотрудник, TalypovAE@sklif.mos.ru

Львов Иван Сергеевич*, к.м.н., старший научный сотрудник. LvovIS@sklif.mos.ru

Кордонский Антон Юрьевич*, к.м.н., старший научный сотрудник. KordonskiyAJ@sklif.mos.ru

Лебедев Валерий Борисович**, к.м.н., врач травматолог-ортопед, horizont_vbl@mail.ru

Ответственные исполнители:

Зуев Сергей Евгеньевич*, научный сотрудник +7 (915) 377-...

---

* НИИ СП им. Н.В. Склифосовского; ** ФГБУ «НМХЦ им. Н.И. Пирогова» Минздрава России; ***ФГБУ «Федеральный центр мозга и нейротехнологий» ФМБА

Сосновский Евгений Александрович, врач-нейрохирург, к.м.н. +7 (926) 572-...
Епифанов Дмитрий Сергеевич, врач-нейрохирург, +7 (965) 302-...
Каландари Алик Амиранович, ведущий научный сотрудник, д.м.н. +7 (925) 484-...

Это согласие было рассмотрено и одобрено этическими комитетами всех участвующих учреждений. Если у Вас возникли вопросы об этических аспектах предстоящего исследования Вы можете связаться с соответствующим комитетом для уточнения информации.

Учреждение:	Адрес и телефон комитета по этике:	Номер одобрения:
НИИ СП им. Н.В. Склифосовского	г. Москва, Б. Сухаревская площадь 3, стр. 5<br>+7 (495) 628-35-02	1-22/11.01.22
ФГБУ «НМХЦ им. Н.И. Пирогова» Минздрава России	г. Москва, ул. Нижняя Первомайская, д. 65, каб. 634<br>+7 (499) 464-03-03, доб. 1232	2/16.02.2022
ФГБУ «Федеральный центр мозга и нейротехнологий» ФМБА	г. Москва, улица Островитянова, 1, стр. 10<br>+7 (495) 280-35-50	01/04-03-22

Часть 2. Информированное согласие на участие в исследовании

Я, (Ф.И.О.)
даю добровольное согласие принять участие в исследовании: «открытое проспективное мультицентровое рандомизированное исследование необходимости выполнения стабилизации позвоночника после декомпрессии невральных структур у пациентов с одноуровневыми стенозами позвоночного канала на поясничном уровне».

Я получил(а) исчерпывающие разъяснения от сотрудника, который обсуждал со мной вопрос о моем участии в исследовании, по поводу характера, целей и продолжительности данного исследования.

Я подтверждаю, что я полностью прочитал(а) и понял(а) прилагаемую информацию. Мне была предоставлена полная и понятная информация для участника исследования. У меня была возможность задать все возникшие вопросы.

Я понимаю, что участие в этом исследовании добровольное. Я могу в любое время и без объяснения причин забрать свое согласие, и это не повлечет никаких нежелательных последствий для моего дальнейшего лечения.

Я понимаю, что уполномоченные представители контролирующих организаций и этического комитета могут ознакомиться с некоторыми разделами моей медицинской документации, относящейся к моему участию в данном исследовании. Своей подписью я предоставляю им право доступа к моей медицинской документации.

Я понимаю, что в ходе данного исследования будет собрана информация, которая будет рассматриваться как конфиденциальная. Никому и никогда не будет сообщаться мое имя.

Я не буду пытаться ограничить возможное использование результатов исследования.

Я согласен(сна) принять участие в данном исследовании и сотрудничать с руководителем и ответственными исполнителями исследования. Я обязуюсь немедленно сообщать обо всех замеченных отклонениях от нормы.

Я согласен (сна) с тем, что мой лечащий врач или другие врачи, ответственные за мое лечение, будут проинформированы о моем участии в данном исследовании.

Я согласен(сна) с тем, что мой врач-исследователь может обратиться к моим родственникам или знакомым, лечащему врачу или другим врачам, ответственным за мое лечение, для получения информации о состоянии моего здоровья, если это важно для данного исследования.

Я получил (а) подписанный экземпляр этой формы информации для пациента и согласия на участие в исследовании.

Пациент:

____

фамилия, имя, отчество подпись дата

Врач:

____

фамилия, имя, отчество подпись Дата

Приложение 2. Описание шкал, применяемых в исследовании

SF-36

ИНСТРУКЦИИ: Этот опросник содержит вопросы, касающиеся Ваших взглядов на свое здоровье. Предоставленная информация поможет следить за тем, как Вы себя чувствуете, и насколько хорошо справляетесь со своими обычными нагрузками. Ответьте на каждый вопрос, помечая выбранный Вами ответ так, как это указано. Если Вы не уверены в том, как ответить на вопрос, пожалуйста, выберите такой ответ, который точнее всего отражает Ваше мнение.

1. В целом Вы оценили бы состояние Вашего здоровья как (обведите одну цифру):

Отличное..1
Очень хорошее.............................2
Хорошее..3
Посредственное..........................4
Плохое...5

2. Как бы Вы в целом оценили свое здоровье сейчас по сравнению с тем, что было год назад? (обведите одну цифру)

Значительно лучше, чем год назад......................1
Несколько лучше, чем год назад...........................2
Примерно так же, как год назад............................3
Несколько хуже, чем год назад..............................4
Гораздо хуже, чем год назад.....................................5

3. Следующие вопросы касаются физических нагрузок, с которыми Вы, возможно, сталкиваетесь в течении своего обычного дня. Ограничивает ли Вас состояние Вашего здоровья в настоящее время в выполнении перечисленных ниже физических нагрузок? Если да, то в какой степени? (обведите одну цифру в каждой строке)

	**Вид физической активности**	**Да, значительно ограничивает**	**Да, немного ограничивает**	**Нет, совсем не ограничивает**
а	Тяжелые физические нагрузки, такие как бег, поднятие тяжестей, занятие силовыми видами спорта	1	2	3
б	Умеренные физические нагрузки, такие как передвинуть стол, поработать с пылесосом, собирать грибы или ягоды	1	2	3
в	Поднять или нести сумку с продуктами	1	2	3
г	Подняться пешком по лестнице на несколько пролетов	1	2	3
д	Подняться пешком по лестнице на один пролет	1	2	3
е	Наклониться, встать на колени, присесть на корточки	1	2	3
ж	Пройти расстояние более одного километра	1	2	3
з	Пройти расстояние в несколько кварталов	1	2	3
и	Пройти расстояние в один квартал	1	2	3
к	Самостоятельно вымыться, одеться	1	2	3

4. Бывало ли за последние 4 недели, что Ваше физическое состояние вызывало затруднения в Вашей работе или другой обычной повседневной деятельности, вследствие чего (обведите одну цифру в каждой строке):

		Да	Нет
а	Пришлось сократить количество времени, затрачиваемого на работу или другие дела	1	2
б	Выполнили меньше, чем хотели	1	2
в	Вы были ограничены в выполнении какого-либо определенного вида работы или другой деятельности	1	2
г	Были трудности при выполнении своей работы или других дел (например, они потребовали дополнительных усилий)	1	2

5. Бывало ли за последние 4 недели, что Ваше эмоциональное состояние вызывало затруднения в Вашей работе или другой обычной повседневной деятельности, вследствие чего (обведите одну цифру в каждой строке):

		Да	Нет
а	Пришлось сократить количество времени, затрачиваемого на работу или другие дела	1	2
б	Выполнили меньше, чем хотели	1	2
в	Выполняли свою работу или другие дела не так аккуратно, как обычно	1	2

6. Насколько Ваше физическое состояние или эмоциональное состояние в течении последних 4 недель мешало Вам проводить время с семьей, друзьями, соседями или в коллективе? (обведите одну цифру)

Совсем не мешало..............................1
Немного...2
Умеренно..3
Сильно...4
Очень сильно..5

7. Насколько сильную физическую боль Вы испытывали за последние 4 недели? (обведите одну цифру)

Совсем не испытывал(а)......................1
Очень слабую..2
Слабую...3
Умеренную..4
Сильную...5
Очень сильную..6

8. В какой степени боль в течение последних 4 недель мешала Вам заниматься Вашей нормальной работой, включая работу вне дома и по дому? (обведите одну цифру)

Совсем не мешало...............................1
Немного...2
Умеренно..3
Сильно..4
Очень сильно..5

9. Следующие вопросы касаются того, как Вы себя чувствовали и каким было Ваше настроение в течение последних 4 недель. Пожалуйста, на каждый вопрос дайте один ответ, который наиболее соответствует Вашим ощущениям. Как часто в течении последних 4 недель (обведите одну цифру в каждой строке):

		Все время	Большую часть времени	Часто	Иногда	Редко	Ни разу
а	Вы чувствовали себя бодрым(ой)?	1	2	3	4	5	6
б	Вы сильно нервничали?	1	2	3	4	5	6
в	Вы чувствовали себя таким(ой) подавленным(ой), что ничто не могло Вас взбодрить?	1	2	3	4	5	6
г	Вы чувствовали себя спокойным(ой) и умиротворенным(ой)?	1	2	3	4	5	6
д	Вы чувствовали себя полным(ой) сил и энергии?	1	2	3	4	5	6
е	Вы чувствовали себя упавшим (ей) духом и печальным(ой)?	1	2	3	4	5	6
ж	Вы чувствовали себя измученным(ой)?	1	2	3	4	5	6
З	Вы чувствовали себя счастливым(ой)?	1	2	3	4	5	6
и	Вы чувствовали себя уставшим (ей)?	1	2	3	4	5	6

10. Как часто в последние 4 недели Ваше физическое или эмоциональное состояние мешало Вам активно общаться с людьми? Например, навещать родственников, друзей и т.п. (обведите одну цифру)

Все время..1
Большую часть времени...............2
Иногда 3
Редко...4
Ни разу...5

11. Насколько ВЕРНЫМ или НЕВЕРНЫМ представляется по отношению к Вам каждое из ниже перечисленных утверждений? (обведите одну цифру в каждой строке)

		Определенно верно	В основном верно	Не знаю	В основном неверно	Определенно неверно
а	Мне кажется, что я более склонен к болезням, чем другие	1	2	3	4	5
б	Мое здоровье не хуже, чем у большинства моих знакомых	1	2	3	4	5
в	Я ожидаю, что мое здоровье ухудшится	1	2	3	4	5
г	У меня отличное здоровье	1	2	3	4	5

Шкала Oswestry

ИНСТРУКЦИИ
Пожалуйста, в каждом разделе дайте один ответ, который наиболее соответствует Вашим ощущениям. Обведите букву необходимого варианта ответа.

Раздел 1. Интенсивность болевого синдрома

А. Я могу переносить боль без приема болеутоляющих препаратов.
Б. Боль сильная, но болеутоляющих препаратов я не принимаю.
В. Болеутоляющие препараты полностью избавляют меня от боли.
Г. Болеутоляющие препараты умеренно снижают боль.
Д. Болеутоляющие препараты слабо уменьшают боль.
Е. Болеутоляющие препараты мне не помогают, поэтому я и не принимаю.

Раздел 2. Самообслуживание (умывание, одевание и т.д.)

А. Самообслуживание не нарушено и не вызывает дополнительной боли.
Б. Самообслуживание не нарушено, но вызывает дополнительную боль.
В. При самообслуживании из-за боли я все делаю медленно.
Г. При самообслуживании я нуждаюсь в некоторой помощи, хотя в основном все делаю самостоятельно.
Д. В большинстве случаев мне необходима помощь.
Е. Самостоятельно я не могу ничего сделать.

Раздел 3. Поднимание предметов

А. Я могу поднимать тяжелые предметы без появления дополнительной боли.
Б. Я могу поднимать тяжелые предметы, но это усиливает боль.
В. Боль мешает мне поднимать тяжелые предметы, но я могу это сделать, если они удобно расположены (например, на столе).
Г. Боль мешает мне поднимать тяжелые предметы, но я могу поднять предмет средней тяжести, если он удобно расположен.
Д. Я могу поднимать только легкие предметы.
Е. Я не могу не поднять, ни удержать никакие предметы.

Раздел 4. Ходьба

А. Боль не мешает мне проходить любые расстояния.
Б. Боль мешает мне пройти больше 1 км.
В. Боль мешает мне пройти больше 500 м.
Г. Боль мешает мне пройти больше 250 м.
Д. Я могу ходить только с опорой (с тростью или с костылями).
Е. В основном я лежу и с трудом добираюсь до туалета.

Раздел 5. Сидение

А. Я могу сидеть на любом стуле сколько угодно.
Б. Я могу сидеть долго только на моем любимом стуле.
В. Боль мешает мне сидеть больше 1 часа.
Г. Боль мешает мне сидеть больше 30 мин.
Д. Боль мешает мне сидеть больше 10 мин.
Е. Из-за боли я вообще не могу сидеть.

Раздел 6. Стояние

А. Я могу стоять сколько угодно без усиления боли.
Б. Я могу стоять сколько угодно, но это усиливает боль.
В. Боль мешает мне стоять больше 1 ч.
Г. Боль мешает мне стоять больше 30 мин.
Д. Боль мешает мне стоять больше 10 мин.
Е. Из-за боли я вообще не могу стоять.

Раздел 7. Сон

А. Сон у меня хороший, боль его не нарушает.
Б. Я испытываю боль, но сплю хорошо.
В. Из-за боли я сплю на 2 часа меньше, чем раньше.
Г. Из-за боли я сплю на 4 часа меньше, чем раньше.
Д. Из-за боли я сплю на 6 часов меньше, чем раньше.
Е. Из-за боли я не сплю вообще.

Раздел 8. Сексуальная жизнь

А. Моя сексуальная жизнь нормальна и не вызывает дополнительной боли.
Б. Моя сексуальная жизнь нормальна, но усиливает боль.
В. Моя сексуальная жизнь нормальна, но резко усиливает боль.
Г. Боль значительно ограничивает мою сексуальную жизнь.
Д. Боль почти полностью препятствует моей сексуальной жизни.
Е. Из-за боли сексуальная жизнь невозможна.

Раздел 9. Общественная жизнь

А. Моя общественная жизнь обычная и не усиливает боли.
Б. Моя общественная жизнь обычная, но усиливает боль.
В. Боль сильно сказывается на моей общественной жизни, но ограничивает лишь те ее области, которые требуют больших затрат сил (например, танцы).
Г. Боль существенно ограничивает мою общественную жизнь, из-за нее я часто не могу выйти из дома.
Д. Боль ограничила мою общественную жизнь только моим домом.
Е. Из-за боли я совсем не участвую в общественной жизни.

Раздел 10. Поездки

А. Я могу ездить, куда захочу без усиления боли.
Б. Я могу ездить куда угодно, но это усиливает боль.
В. Боль мешает поездкам длительностью более 1 часа.
Г. Из-за боли я могу совершать поездки длительностью не более 30 минут.
Д. Из-за боли я могу совершать поездки только на определенном транспорте.
Е. Я могу ехать куда-то только лежа.

EQ-5D-5L

В каждом разделе отметьте галочкой ОДИН квадратик, который наилучшим образом отражает состояние Вашего здоровья СЕГОДНЯ.

ПОДВИЖНОСТЬ

Я не испытываю никаких трудностей при ходьбе ☐
Я испытываю небольшие трудности при ходьбе ☐
Я испытываю умеренные трудности при ходьбе ☐
Я испытываю большие трудности при ходьбе ☐
Я не в состоянии ходить ☐

УХОД ЗА СОБОЙ

Я не испытываю никаких трудностей с мытьем или одеванием ☐
Я испытываю небольшие трудности с мытьем или одеванием ☐
Я испытываю умеренные трудности с мытьем или одеванием ☐
Я испытываю большие трудности с мытьем или одеванием ☐
Я не в состоянии сам (-а) мыться или одеваться ☐

ПРИВЫЧНАЯ ПОВСЕДНЕВНАЯ ДЕЯТЕЛЬНОСТЬ *(например: работа, учеба, работа г дому, участие в делах семьи, досуг)*

Моя привычная повседневная деятельность дается мне без труда ☐
Моя привычная повседневная деятельность для меня немного затруднительна ☐
Моя привычная повседневная деятельность для меня умеренно затруднительна ☐
Моя привычная повседневная деятельность для меня очень затруднительна ☐
Я не в состоянии заниматься своей привычной повседневной деятельностью ☐

БОЛЬ/ДИСКОМФОРТ

Я не испытываю боли или дискомфорта ☐
Я испытываю небольшую боль или дискомфорт ☐
Я испытываю умеренную боль или дискомфорт ☐
Я испытываю сильную боль или дискомфорт ☐
Я испытываю чрезвычайно сильную боль или дискомфорт ☐

ТРЕВОГА/ДЕПРЕССИЯ

Я не испытываю тревоги или депрессии ☐
Я испытываю небольшую тревогу или депрессию ☐
Я испытываю умеренную тревогу или депрессию ☐
Я испытываю сильную тревогу или депрессию ☐
Я испытываю крайне сильную тревогу или депрессию ☐

Russia (Russian) © 2009 EuroQol Group. EQ-5D™ is a trade mark of the EuroQol Group

Наилучшее состояние здоровья, которое можно себе представить

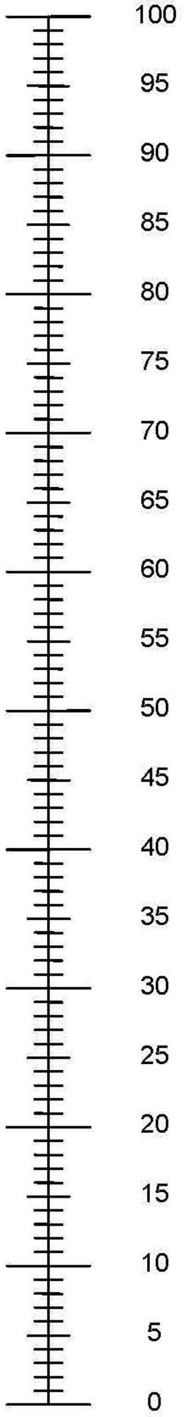

Наихудшее состояние здоровья, которое можно себе представить

- Мы хотели бы узнать, как бы Вы оценили состояние своего здоровья СЕГОДНЯ.
- Перед Вами шкала от 0 до 100.
- 100 означает наилучшее состояние здоровья, которое можно себе представить,
  0 – наихудшее состояние здоровья, которое можно себе представить.
- Поставьте крестик “X” на шкале в том месте, которое, по Вашему мнению, соответствует состоянию Вашего здоровья СЕГОДНЯ.
- Теперь впишите отмеченное Вами на шкале число в приведенный ниже квадрат.

СОСТОЯНИЕ ВАШЕГО ЗДОРОВЬЯ СЕГОДНЯ = ☐

Russia (Russian) © 2009 EuroQol Group. EQ-5D™ is a trade mark of the EuroQol Group

Шкала боли VAS

Обозначьте цифру, которой соответствует, по вашему мнению, выраженность болей в шее

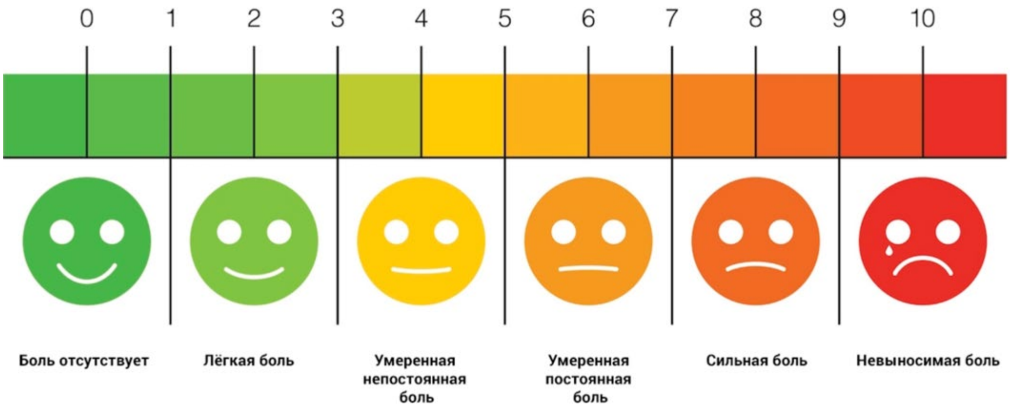

Шкала совладания с болью (PCI F.W. Kraaimaat and A.W.M. Evers)

Люди, страдающие от боли, разрабатывают различные способы управления этой болью. На следующих страницах приводится ряд утверждений о том, что вы делаете или думаете, когда испытываете боль.

Мы просим Вас указать, как часто вы действуете или думаете каждым из описываемых способов. Сделайте это, зачеркнув один из возможных вариантов.

Не торопитесь, здесь нет правильных или неправильных ответов: важно ваше мнение. Само собой разумеется, что не все утверждения применимы к вам.

1. Я бросил свою деятельность.

   1 – редко 3 – часто
   2 - иногда 4 – очень часто

2. Я продолжаю свою деятельность, но с меньшими усилиями

   1 – редко 3 – часто
   2 - иногда 4 – очень часто

3. Я продолжаю свою деятельность, но в более медленном темпе

   1 – редко 3 – часто
   2 - иногда 4 – очень часто

4. Я продолжаю свою деятельность, но уже менее точно.

   1 – редко 3 – часто
   2 - иногда 4 – очень часто

5. Я ограничиваюсь простыми делами.

   1 – редко 3 – часто
   2 - иногда 4 – очень часто

6. Я забочусь о том, чтобы мне не приходилось напрягаться физически.

   1 – редко 3 – часто
   2 - иногда 4 – очень часто

7. Я отдыхаю, сидя или лежа.

   1 – редко 3 – часто
   2 - иногда 4 – очень часто

8. Я принимаю удобную позу тела

   1 – редко 3 – часто
   2 - иногда 4 – очень часто

9. Я принимаю ванну или душ

   1 – редко 3 – часто
   2 - иногда 4 – очень часто

10. Я забочусь о том, чтобы не расстраиваться

   1 – редко 3 – часто
   2 - иногда 4 – очень часто

11. Я отступаю в спокойной обстановке.

   1 – редко 3 – часто
   2 - иногда 4 – очень часто

12. Я избегаю беспокоящих звуков.

   1 – редко 3 – часто
   2 - иногда 4 – очень часто

13. Я избегаю яркого света (т.е. надеваю солнцезащитные очки, задергиваю шторы).

   1 – редко 3 – часто
   2 - иногда 4 – очень часто

14. Я забочусь о том, что я ем или пью

   1 – редко 3 – часто
   2 - иногда 4 – очень часто

15. Я притворяюсь, что боли нет

   1 – редко 3 – часто
   2 - иногда 4 – очень часто

16. Я делаю вид, что боль не касается моего тела

   1 – редко 3 – часто
   2 - иногда 4 – очень часто

17. Я все время сосредотачиваюсь на боли.

   1 – редко 3 – часто
   2 - иногда 4 – очень часто

18. Я представляю себе боль менее жестокой, чем она есть на самом деле.

   1 – редко 3 – часто
   2 - иногда 4 – очень часто

19. Я думаю о приятных вещах или событиях.

1 – редко 3 – часто
2 - иногда 4 – очень часто

20. Я отвлекаюсь занимаюсь физическими упражнениями (например, ходьба, езда на велосипеде или плавание)

1 – редко 3 – часто
2 - иногда 4 – очень часто

21. Я сам читаю, слушаю музыку, смотрю телепрограмму или что-то в этом роде

1 – редко 3 – часто
2 - иногда 4 – очень часто

22. Я делаю то, что нахожу приятным.

1 – редко 3 – часто
2 - иногда 4 – очень часто

23. Я управляю другими физическими стимулами (например, сжимая кулаки, щипая себя, нажимая или потирая место боли).

1 – редко 3 – часто
2 - иногда 4 – очень часто

24. Я думаю о вещах, которые не могу выполнить из-за боли.

1 – редко 3 – часто
2 - иногда 4 – очень часто

25. Я начинаю волноваться.

1 – редко 3 – часто
2 - иногда 4 – очень часто

26. Я задаюсь вопросом о причине боли.

1 – редко 3 – часто
2 - иногда 4 – очень часто

27. Я думаю, что боль будет усиливаться.

1 – редко 3 – часто
2 - иногда 4 – очень часто

28. Я вспоминаю моменты, когда мне не было больно.

1 – редко 3 – часто
2 - иногда 4 – очень часто

29. Мне кажется, я схожу с ума от боли.

1 – редко 3 – часто
2 - иногда 4 – очень часто

30. Я думаю о трудностях других людей.

1 – редко 3 – часто
2 - иногда 4 – очень часто

31. Другие не понимают, что такое испытывать такую боль.

1 – редко 3 – часто
2 - иногда 4 – очень часто

32. Я отделяюсь от себя.

1 – редко 3 – часто
2 - иногда 4 – очень часто

33. Если я выхожу из дома, я стараюсь вернуться как можно скорее

1 – редко 3 – часто
2 - иногда 4 – очень часто

34. у меня есть свой собственный способ уменьшить боль или сделать ее более терпимой.

1 – редко 3 – часто
2 - иногда 4 – очень часто

Опросник Вон Корффа

Инструкция: Вам предложено несколько вопросов о том, какую боль Вы испытываете. Для ответа используйте промежуточные значения шкалы от 0 до 10 баллов, где **0 баллов = отсутствие боли**, а **10 баллов = самая сильная боль**, какая только могла бы быть. В вопросе №4 укажите количество дней.

№	Вопрос.	Баллы
1.	Как бы Вы оценили интенсивность боли **в настоящий момент** по шкале от 0 до 10?	
2.	На сколько баллов Вы бы оценили самую **сильную** боль за **последние 6** месяцев?	
3.	На сколько баллов Вы бы оценили **среднюю интенсивность** боли за **последние 6** месяцев?	
4.	Сколько дней за последние 6 месяцев Вы были не в состоянии справляться со своими обычными делами (работа, учеба, домашние обязанности) из-за боли?	Количество дней:
5.	Как сильно за последние 6 месяцев боль мешала вашей повседневной активности?	
6.	Как часто за последние 6 месяцев боль заставляла вас отказываться от проведения досуга с семьей или участия в культурных мероприятиях?	
7.	Насколько сильно за последние 6 месяцев боль повлияла на вашу работоспособность (включая домашние дела) ?	

Шкала катастрофизации боли

У всех бывают обстоятельства в какой-либо момент жизни, когда испытываешь боль. Такие обстоятельства могут включать головную, зубную боли, боль в суставах или мышцах. Люди часто испытывают боль при таких обстоятельствах, как болезнь, травма, стоматологические процедуры или хирургическое вмешательство.

Нас интересует, какие мысли и чувства у Вас возникают, когда Вы испытываете боль. Ниже даны тринадцать утверждений, описывающие различные мысли и чувства, связанные с болью. С помощью данной шкалы, пожалуйста, укажите, в какой степени у Вас бывают такие чувства и мысли, когда Вы испытываете боль

1. Я все время переживаю, пройдет ли боль

0 – совсем нет 3 - сильно
1 - немного 4 – все время
2 - умеренно

2. Я чувствую, что так больше не может продолжаться

0 – совсем нет 3 - сильно
1 - немного 4 – все время
2 - умеренно

3. Это ужасно, и я думаю, что лучше никогда не станет

0 – совсем нет 3 - сильно
1 - немного 4 – все время
2 - умеренно

4. Это ужасно, и я чувствую, что она меня порабощает

0 – совсем нет 3 - сильно
1 - немного 4 – все время
2 - умеренно

5. Я чувствую, что не могу это терпеть

0 – совсем нет 3 - сильно
1 - немного 4 – все время
2 - умеренно

6. Мне становится страшно, что боль усилится

0 – совсем нет 3 - сильно
1 - немного 4 – все время
2 - умеренно

7. Я все время думаю о других эпизодах боли

0 – совсем нет 3 - сильно
1 - немного 4 – все время
2 - умеренно

8. Я очень сильно хочу, чтобы боль прошла

0 – совсем нет 3 - сильно
1 - немного 4 – все время
2 - умеренно

9. Мне кажется, я никак не могу перестать думать о ней

0 – совсем нет 3 - сильно
1 - немного 4 – все время
2 - умеренно

10. Я все время думаю о том, как же мне больно

0 – совсем нет 3 - сильно
1 - немного 4 – все время
2 - умеренно

11. Я все время думаю о том, как сильно хочу, чтобы боль ушла

0 – совсем нет 3 - сильно
1 - немного 4 – все время
2 - умеренно

12. Я ничего не могу сделать, чтобы уменьшить боль

0 – совсем нет 3 - сильно
1 - немного 4 – все время
2 - умеренно

13. Я задумываюсь над тем, может ли это быть что-то серьезное

0 – совсем нет 3 - сильно
1 - немного 4 – все время
2 - умеренно
